# Low-Dose Aspirin for Cardiovascular Disease Primary Prevention in Patients With Giant Cell Arteritis

**DOI:** 10.1001/jamanetworkopen.2026.6579

**Published:** 2026-04-17

**Authors:** Maxime Beydon, David Hajage, Alexis F. Guédon, Fabrice Carrat, Raphaèle Seror, Florence Tubach

**Affiliations:** 1 Sorbonne Université, Institut pour la Santé et la Recherche Médicale, Institut Pierre Louis d’Epidémiologie et de Santé Publique, Equipe PEPITES, Assistance Publique - Hôpitaux de Paris, Hôpital Pitié Salpêtrière, Département de Santé Publique, Centre de Pharmacoépidémiologie (Cephepi), Paris, France; 2 Department of Internal Medicine, Hôpital Saint-Antoine, Assistance Publique - Hôpitaux de Paris, Sorbonne Université, Paris, France; 3 Sorbonne Université, Institut National de la Santé et de la Recherche Médicale, Institut Pierre Louis d’Epidémiologie et de Santé Publique, Département de santé publique, Hôpital Saint-Antoine, Assistance Publique - Hôpitaux de Paris, Paris, France; 4 Department of Rheumatology, Hôpital Bicêtre, Assistance Publique - Hôpitaux de Paris, Université Paris-Saclay, Le Kremlin-Bicêtre, France; 5 Center for Immunology of Viral Infections and Auto-immune Diseases, Institut pour la Santé et la Recherche Médicale UMR 1184, Université Paris-Saclay, Le Kremlin-Bicêtre, Paris, France

## Abstract

**Importance:**

Patients with giant cell arteritis (GCA) face an increased risk of major adverse cardiovascular events (MACE). The benefit of low-dose aspirin in these patients is unknown.

**Objective:**

To evaluate the 1-year association of low-dose aspirin with risk of MACE in primary prevention in patients with incident GCA and to evaluate the risk of major hemorrhage and net clinical benefit at predefined time points.

**Design, Setting, and Participants:**

This population-based cohort study used a target trial emulation framework with a cloning, censoring, and weighting approach within the French National Health Data System. Participants were individuals aged at least 50 years with incident GCA between 2010 and 2022, without previous cardiovascular events or antiplatelet or anticoagulant use at GCA diagnosis. Analysis was conducted from November 2024 to June 2025.

**Exposure:**

Initiation of low-dose aspirin vs not (control group) within 14 days of GCA diagnosis.

**Main Outcomes and Measures:**

The main outcome was MACE, a composite end point of ischemic stroke, myocardial infarction, and all-cause mortality. Major hemorrhages were evaluated as secondary outcomes.

**Results:**

A total of 14 528 individuals (median [IQR] age, 74 [67 to 80] years; 10 396 [72%] female), were included. Low-dose aspirin was initiated in 5220 individuals (36%). At 1 year, MACE risk was lower in the low-dose aspirin group (relative risk [RR], 0.86 [95% CI, 0.75 to 0.96]; risk difference [RD], −0.54% [95% CI, −0.99% to −0.12%]), while major hemorrhage risk was higher (RR, 1.29 [95% CI, 1.05 to 1.53]; RD, 0.51% [95% CI, 0.13% to 0.91%]). All-cause mortality was lower in the low-dose aspirin group at 1 year (RD, −0.43% [95% CI, −0.77% to −0.10%]). At 3 years, MACE events were less frequent in the low-dose aspirin group (RD, −1.08% [95% CI, −1.77% to −0.41%]), with no difference in major hemorrhages. A more pronounced association between low-dose aspirin and lower 1-year MACE was observed in women (RD, −0.78% [95% CI, −1.29% to −0.25%]) and patients with diabetes at GCA diagnosis (RD, −2.23% [95% CI, −3.48% to −1.02%]).

**Conclusions and Relevance:**

In this retrospective cohort study, low-dose aspirin at GCA diagnosis was associated with lower MACE at 1 and 3 years but higher hemorrhage risk at 1 year. Subgroup analyses suggested heterogeneity according to sex and diabetic status.

## Key Points

**Question:**

Is low-dose aspirin initiation in patients without cardiovascular disease at giant cell arteritis (GCA) diagnosis associated with risk of cardiovascular events?

**Findings:**

In this this cohort study including 14 528 patients, with 36% initiating low-dose aspirin at GCA diagnosis, low-dose aspirin was associated with reduced risk of major cardiovascular events, including all-cause mortality, at 1 year but with an increased risk of major hemorrhage. The association between low-dose aspirin and reduced cardiovascular risk was more pronounced in women and in patients with diabetes.

**Meaning:**

These findings suggest that low-dose aspirin initiation at GCA diagnosis was associated with lower risk of cardiovascular events but with increased hemorrhagic risk.

## Teaser

This cohort study uses a target trial emulation framework to examine whether initiation of low-dose aspirin at giant cell arteritis diagnosis is associated with reduced risk of major adverse cardiovascular events.

## Introduction

Giant cell arteritis (GCA) is a large-vessel vasculitis that typically affects individuals older than 70 years and predominantly involves the supra-aortic vessels. As in other inflammatory diseases,^1^ patients with GCA face an elevated risk of cardiovascular disease (CVD). This risk is highest in the months following GCA diagnosis, when vascular inflammation is maximal and exposure to corticosteroids is greatest,^2,3^ but it persists thereafter. Based on small, retrospective studies,^4,5^ European guidelines recommended in 2009 systematic use of low-dose aspirin at GCA diagnosis unless contraindicated.^6^ A more recent meta-analysis from 2014 found no evidence supporting the use of low-dose aspirin in GCA.^7^ Consequently, the latest 2018 guidelines no longer endorse the routine use of antiplatelet therapy in GCA unless otherwise indicated.^8^ Despite these updates, prescribing aspirin for primary CVD prevention in GCA remains a common practice, with French guidelines advocating for its prescription in case of ischemic risk.^9^

More broadly, outside the context of GCA, large trials have shown that aspirin does not reduce the incidence of major adverse cardiovascular events (MACE) in elderly populations without prior CVD, while increasing the risk of bleeding and possibly mortality.^10,11,12^ However, European Society of Cardiology guidelines from 2021 identified a gap in cardiovascular risk assessment for inflammatory diseases,^13^ questioning the applicability of these findings to GCA.

Since GCA is a rare disease, conducting a randomized trial focusing on primary cardiovascular prevention in this population would be difficult to achieve, costly, and time-consuming. When randomized trial data are unavailable, observational analyses using a target trial emulation approach may be used to guide clinical practice. To generate a first piece of evidence, we proposed to emulate a target trial using a retrospective cohort from the nationwide French National Health Data System (Système National des Données de Santé [SNDS]). We hypothesized low-dose aspirin could decrease risk of MACE but would increase hemorrhagic risk. The primary objective of our study was to evaluate the association of initiation of low-dose aspirin with primary prevention of MACE at 1 year in patients with incident GCA. Secondary objectives include assessing earlier and longer-term evaluation of MACE, the occurrence of major hemorrhagic events, the net clinical benefit, and specific cardiovascular end points.

## Methods

### Target Trial Emulation

This cohort study was authorized under decree 2016-1871 relating to the processing of personal data from the SNDS and French law (articles 1461-1 and 1461-2). The Assistance Publique-Hôpitaux de Paris, as an authorized permanent user of the SNDS, maintains an internal registry for SNDS-based research; the study was declared to this registry, which serves as the institutional equivalent of ethics committee approval. Informed consent was waived as the study relied exclusively on routinely collected administrative data. To evaluate the association between low-dose aspirin in primary prevention and CVD in individuals with incident GCA, we applied the target trial emulation framework.^14^ Full details on the target trial and its emulation are available in eTable 1 in Supplement 1. We followed the Reporting of Studies Conducted Using Observational Routinely Collected Health Data Statement for Pharmacoepidemiology (RECORD-PE) reporting guidelines^15^ as well as principals of the Transparent Reporting of Observational Studies Emulating a Target Trial (TARGET) guidelines.^16^

### Data Source

We designed a retrospective cohort within the SNDS, which links the health insurance claims database, the hospital discharge database and the death certificates database. It provides quasiexhaustive nationwide data for the population of France (approximately 67 million individuals). This database is now routinely used for research purposes,^17,18^ and some studies have shown the reliability of in-hospital diagnostic codes for cardiovascular outcomes and hemorrhagic events.^19,20^ Data are pseudonymized, and insured individuals are informed that their data may be reused for research and retain rights of access, rectification, and opposition to reuse.

### Patients

Eligible patients were individuals with a first hospitalization for GCA between January 1, 2010, and December 31, 2022, as patients with suspected GCA were routinely hospitalized in France during the study period. Positive predictive value of GCA diagnostic codes in the hospital discharge database have been shown to be excellent.^21^ The minimum look-back period was 3 years.

Participants were aged at least 50 years, alive at discharge, had an index hospitalization lasting no more than 28 days, and had no history of cardiovascular events (ie, myocardial infarction or revascularization, transient or established stroke, peripheral artery disease, or carotid artery stenosis), atrial fibrillation, or prior or concurrent anterior ischemic optic neuropathy at index hospitalization. Patients with any dispensing of antiplatelet or anticoagulant therapy within 3 months before GCA diagnosis, those residing in residential care facilities, and those with any corticosteroid dispensing requirement after discharge were excluded to avoid postbaseline selection bias.

### Exposure and Treatment Allocation

The exposure was initiation of low-dose aspirin (75 to 300 mg) at discharge of index hospitalization, as determined by pharmacy dispensing data. To account for potential delay between hospital discharge and pharmacy dispensing, a 14-day window (ie, grace period) was allowed for treatment allocation. Control patients were patients without dispensing of low-dose aspirin during the grace period.

### Follow-Up and Outcomes

Patients were followed-up through December 31, 2023, ensuring at least 1 year of follow-up for all. The primary outcome was any MACE, defined as incident acute myocardial infarction, stroke, or death from any cause at 1 year. Secondary outcomes included additional time points (6 months, 2 years, and 3 years), individual components of the primary outcome, major hemorrhage (hospitalization for digestive, intracranial, or other bleeding as the primary diagnosis), and a composite of MACE and major hemorrhage, defined as net clinical benefit, in line with randomized trials.^22,23^ We also examined a composite cardiovascular outcome including myocardial infarction, coronary revascularization, stroke, limb ischemia or revascularization, and cardiovascular mortality, as well as each component individually, and assessed incident anterior optic ischemic neuropathy. Diagnostic codes are provided in eTable 2 in Supplement 1.

### Covariates

We adjusted for potential confounders using inverse probability of censoring weights. The included covariates were sociodemographic characteristics (age at GCA diagnosis, sex, socioeconomic status based on the French Deprivation Index^24^ and full medical expense coverage), CVD risk factors (hypertension, diabetes, dyslipidemia, proxy for tobacco exposure, obesity), comorbidities (falls, fractures, polymedication in the year prior to GCA diagnosis, chronic kidney disease, cardiac insufficiency, cancer, dementia, current or past iron deficiency, history of major hemorrhage, proxy for chronic alcohol abuse, and index hospitalization duration), and GCA-related variables (code for polymyalgia rheumatica at index hospitalization or suspected prevalent polymyalgia rheumatica, defined as >2 dispensed corticosteroid prescription in the year prior to GCA hospitalization; cumulative corticosteroid dose; tocilizumab and methotrexate prescription; and year of diagnosis). Covariate identification is detailed eTable 2 in Supplement 1.

### Statistical Analysis

Descriptive analysis for continuous variables is presented as medians and IQRs. Discrete variables are expressed as numbers and percentages. To address immortal time bias during the 14-day grace period, we applied the cloning-censoring-weighting method.^14,25^ Briefly, all observations were duplicated at baseline, with each clone assigned to 1 of the allocation groups (low-dose aspirin or control). Clones were censored no later than the final day of the grace period if they deviated from their allocated group (eg, low-dose aspirin initiation in the control group or no aspirin initiation by day 14 in the low-dose aspirin group). Events that occurred prior to the censoring of either clone were attributed to both. To account for confounding bias, uncensored observations were upweighted using inverse probability of censoring weights, with daily probability of remaining uncensored during the grace period estimated with a multivariable Cox model.

In the observational analogue of intention-to-treat (ITT) analysis, follow-up in each treatment group continued without additional censoring beyond the grace period in case of deviation from treatment allocation group. In the per-protocol analysis, patients were censored after the grace period if low-dose aspirin was introduced in the control group (unless otherwise indicated by a CVD event) or if aspirin use was interrupted for more than 90 days in the low-dose aspirin group (unless due to a major hemorrhage). Censoring also occurred if another antiplatelet or anticoagulant was prescribed in either group or if patients entered a residential home facility. Uncensored individuals were upweighted accordingly. Full details on weight models are available in eTable 3 and eTable 4 in Supplement 1.

Between-group differences were expressed as risk differences (RDs) at previously defined time points. Cumulative probabilities at these time points were estimated using weighted Kaplan-Meier estimators in each arm, with RDs defined as the difference in event cumulative probabilities between the low-dose aspirin and control groups, and relative risks (RRs) were defined as the ratio between these probabilities. Difference in restricted survival time across groups was also estimated. For outcomes that did not include all-cause mortality, Aalen-Johansen estimators were used to account for the competing risk of death. We also derived number needed to treat (NNT) and number needed to harm (NNH) from these RDs. We calculated 95% CIs using the bootstrap method with 1000 replications. To explore residual confounding regarding the association of low-dose aspirin with MACE and hemorrhagic risk, we investigated a negative control outcome (infections) and a control intervention (laxatives) (eAppendix in Supplement 1).

Results were stratified by sex, age, number of CVD risk factors, diabetes, and combination of sex and age. Multiple sensitivity analyses were performed: excluding patients with more than 2 dispensed corticosteroid prescriptions in the year preceding GCA index hospitalization or any dispensed methotrexate or tocilizumab during or before index hospitalization, censoring patients at 30 days if they had no corticosteroid dispensing by the end of this grace period in either group, excluding patients with any history of low-dose aspirin dispensed before index date, reducing the grace period to 7 days, applying in the per-protocol analysis a 1-month carry-over period after low-dose aspirin interruption for bleeding events, and restricting analyses to individuals with an index date after January 1, 2016.

Statistical significance was determined as 95% CIs that did not cross the null. Analyses were conducted using R software version 4.3.3 (R Project for Statistical Computing) from November 2024 to June 2025.

## Results

### Population

Of 29 068 eligible patients, 14 528 patients (median [IQR} age, 74 [67 to 80] years; 10 396 [72%] female) were included (eFigure 1 in Supplement 1). By the end of individual follow-up, 13 382 patients (92%) had at least 1 corticosteroid dispensing after index date. A total of 5220 patients (36%) initiated low-dose aspirin within the 14 days following hospital discharge and 9269 patients (64%) did not. Most baseline characteristics were similar across treatment groups before weighting, particularly regarding cardiovascular risk factors (Table 1; eTable 5 in Supplement 1). After weighting, all covariates were well-balanced across groups at the end of the grace period (Table 1; eFigure 2 in Supplement 1). The proportion of patients in the low-dose aspirin group increased from 2010 to 2014 and remained stable thereafter (eFigure 3 in Supplement 1). Adherence to allocation group is presented in eFigure 4 in Supplement 1. Among low-dose aspirin initiators, the median (IQR) duration of treatment was 469 (172-1061) days, with 4344 patients (83%) receiving a dose of 75 mg/d and 5201 patients (>99%) receiving doses of 160 mg/d or less.

**Table 1.** Patients’ Characteristics at Baseline and at the End of the Grace Period

Characteristic	Patients, No. (%)				
	All patients at baseline (n = 14 528)	End of grace period			
		Aspirin [n = 5220 (36%)]	Control [n = 9269 (64%)]	SMD	Weighted SMD
Sex					
Women	10 396 (72)	3669 (70)	6702 (72)	0.02	0.002
Men	4132 (28)	1551 (30)	2567 (28)	0.02	0.002
Age at diagnosis, median (IQR), y	74 (67 to 80)	74 (67 to 79)	74 (67 to 80)	0.015	0.003
Polymyalgia rheumatica *ICD-10* code^a^	3362 (23)	1068 (20)	2286 (25)	0.042	0.001
Length of hospital stay at diagnosis, median (IQR), d	6 (3 to 10)	7 (4 to 10)	6 (2 to 10)	−0.145	−0.011
Comorbidities					
Diabetes	2233 (15)	896 (17)	1369 (15)	−0.017	−0.002
Hypertension	8263 (57)	3059 (59)	5241 (57)	−0.012	−0.005
Dyslipidemia	5239 (36)	1936 (37)	3330 (36)	−0.021	−0.010
Cancer	2012 (14)	697 (13)	1326 (14)	0.012	<0.001
Neurocognitive disorder	602 (4.1)	177 (3.4)	431 (4.6)	0.018	0.003
Tobacco use	1634 (11)	640 (12)	1006 (11)	−0.009	0.001
Obesity	574 (4.0)	209 (4.0)	367 (4.0)	0.004	0.002
Chronic kidney disease	355 (2.4)	113 (2.2)	246 (2.7)	0.007	0.003
Alcohol abuse	287 (2.0)	114 (2.2)	176 (1.9)	−0.003	<0.001
Cardiac insufficiency	185 (1.3)	63 (1.2)	123 (1.3)	0.007	0.003
History of major hemorrhage	553 (3.8)	176 (3.4)	376 (4.1)	0.007	<0.001
Actual or past iron deficiency	2978 (20)	1036 (20)	1975 (21)	0.015	−0.001
Treatments					
Frequent corticosteroid use^b^	2774 (19)	753 (14)	2014 (22)	0.073	0.009
Tocilizumab	121 (0.8)	79 (1.5)	131 (1.4)	−0.001	<0.001
Methotrexate	419 (2.9)	162 (3.1)	318 (3.4)	0.003	0.001
Polymedication^c^	4301 (30)	1335 (26)	2955 (32)	0.063	0.006
History of low-dose aspirin use	1213 (8.3)	456 (8.7)	749 (8.1)	−0.006	−0.003
Current proton pump inhibitor use^d^	7422 (51)	3647 (70)	5011 (54)	−0.158	0.003

Abbreviations: *ICD-10*, *International Statistical Classification of Diseases and Related Health Problems, Tenth Revision*; SMD, standardized mean differences.

^a^ Polymyalgia rheumatica was identified through International Classification of disease 10 codes at index hospitalization.

^b^ Defined as more than 2 corticosteroid dispensing in the year preceding giant cell arteritis index hospitalization.

^c^ Defined as at least 5 distinct drugs dispensed at least 3 times in the year preceding giant cell arteritis index hospitalization.

^d^ Defined as any proton pump inhibitor in the 3 months before the index date (baseline) or in the 3 months before the index hospitalization and up to the end of the grace period.

### Risk of MACE and Secondary Cardiovascular Events: ITT Analogue Analysis

**Figure 1.**
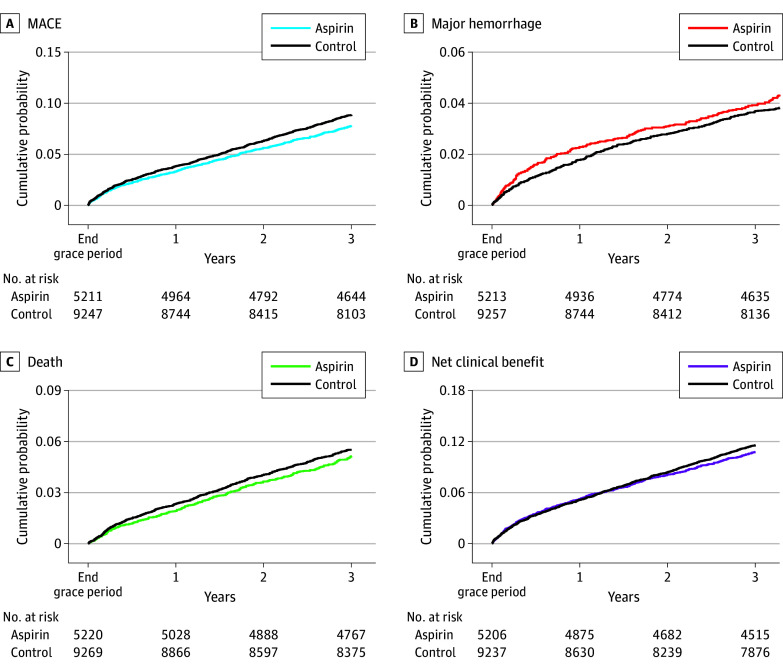
Kaplan-Meier Graphs of Cumulative Incidence of Main and Secondary Outcomes MACE indicates major cardiovascular events (composite outcome of all-cause mortality, myocardial infarction, and ischemic stroke). Major hemorrhage is a composite event of intracranial bleeding, digestive bleeding, and hospitalization for other hemorrhage.

**Figure 2.**
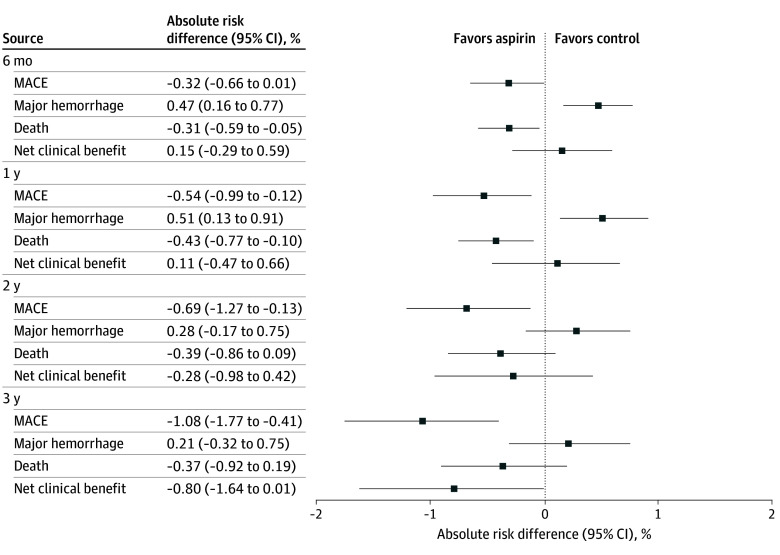
Dot Plot of Risk Differences for Main and Secondary Outcomes MACE indicates major cardiovascular events (composite outcome of all-cause mortality, myocardial infarction, and ischemic stroke). Major hemorrhage is a composite event of intracranial bleeding, digestive bleeding, and hospitalization for other hemorrhage. Risk difference is expressed as the difference in probability between the low-dose aspirin group and the control group.

The 1-year MACE probability was lower in the low-dose aspirin group than the control group (RR, 0.86 [95% CI, 0.75% to 0.96%]; RD, −0.54% [95% CI, −0.99 to −0.12]) (Figure 1). We also observed a lower 1-year all-cause mortality risk in the low-dose aspirin group compared with the control group (RR 0.82 [95% CI, 0.68 to 0.95]; RD, −0.43% (95% CI, −0.77% to −0.10%), while no significant differences were observed for myocardial infarction or stroke (Table 2). The NNT to avoid a MACE at 1 year was 184 and to avoid 1 death was 232. At 1 year, we did not observe any statistically significant difference in coronary events, cardiovascular death, or lower-limb ischemia, nor in the composite outcome of cardiovascular events (eTable 6 in Supplement 1). RDs at 6 months and 2 years are presented in Figure 2 and eTable 7 and eFigure 5 in Supplement 1. Differences in restricted mean survival time are shown in eTable 8 in Supplement 1.

**Table 2.** Cumulative Probability, Risk Difference, and Relative Risk of Primary and Secondary Outcomes at 1 Year and 3 Years

Event	1 y				3 y			
	Events, No. (%)		Absolute difference, % (95% CI)	Relative risk (95% CI)^a^	Events, No. (%)		Absolute difference, % (95% CI)	Relative risk (95% CI)^a^
	Low-dose aspirin	Control			Low-dose aspirin	Control		
MACE^b^	262 (3.28)	454 (3.82)	−0.54 (−0.99 to −0.12)	0.86 (0.75 to 0.96)	490 (7.79)	926 (8.86)	−1.08 (−1.77 to −0.41)	0.88 (0.80 to 0.95)
All-cause mortality	179 (1.91)	339 (2.34)	−0.43 (−0.77 to −0.10)	0.82 (0.68 to 0.95)	341 (5.14)	647 (5.51)	−0.37 (−0.92 to 0.19)	0.93 (0.83 to 1.03)
Myocardial infarction	37 (0.58)	70 (0.66)	−0.08 (−0.28 to 0.12)	0.88 (0.57 to 1.18)	78 (1.34)	175 (1.87)	−0.53 (−0.84 to −0.19)	0.72 (0.56 to 0.88)
Stroke	71 (0.90)	102 (1.03)	−0.13 (−0.36 to 0.09)	0.87 (0.65 to 1.08)	106 (1.69)	193 (2.07)	−0.38 (−0.73 to −0.04)	0.82 (0.65 to 0.97)
Major hemorrhage								
Composite^c^	127 (2.29)	177 (1.78)	0.51 (0.13 to 0.91)	1.29 (1.05 to 1.53)	203 (3.92)	358 (3.71)	0.21 (−0.32 to 0.75)	1.06 (0.91 to 1.20)
Digestive hemorrhage	75 (1.45)	98 (1.01)	0.44 (0.13 to 0.75)	1.44 (1.07 to 1.78)	107 (2.12)	185 (1.95)	0.17 (−0.21 to 0.54)	1.09 (0.88 to 1.28)
Intracranial hemorrhage	29 (0.47)	37 (0.38)	0.09 (−0.08 to 0.25)	1.22 (0.70 to 1.70)	54 (0.94)	72 (0.74)	0.19 (−0.04 to 0.43)	1.26 (0.90 to 1.60)
Other hemorrhage	53 (0.98)	57 (0.55)	0.43 (0.19 to 0.68)	1.80 (1.21 to 2.33)	76 (1.56)	131 (1.33)	0.23 (−0.10 to 0.56)	1.17 (0.90 to 1.43)
Net clinical benefit	344 (5.24)	557 (5.13)	0.11 (−0.47 to 0.66)	1.02 (0.90 to 1.13)	628 (10.80)	1154 (11.60)	−0.80 (−1.64 to 0.01)	0.93 (0.86 to 1.00)

Abbreviation: MACE, major adverse cardiovascular events.

^a^ Risk difference is given by the difference in probability in the low-dose aspirin arm and the control arm.

^b^ The primary outcome, MACE, was a composite outcome of all-cause mortality, myocardial infarction, and stroke.

^c^ Major hemorrhage is a composite of intracranial bleeding, digestive bleeding, and hospitalization for other hemorrhage.

At 3 years, we observed a lower risk of MACE in the low-dose aspirin group (RR, 0.88 [95% CI, 0.80 to 0.95]; RD, −1.08% [95% CI, −1.77% to −0.41%]). In particular, stroke and myocardial infarctions were significantly less frequent in the low-dose aspirin group, but there was no difference for all-cause mortality (Table 2). The NNT to prevent 1 MACE was 93. Myocardial infarction or coronary revascularization occurred less often in the low-dose aspirin group than the control group (RD, −0.65% [95% CI, −1.02 to −0.27]), whereas lower-limb ischemia (RD, 0.56% [95% CI, 0.24% to 0.81%]) and anterior optic ischemic neuropathy (RD, 0.29% [95% CI, 0.11% to 0.49%]) were more frequent, although the absolute number of anterior optic ischemic neuropathy events remained low (77 events over 3 years).

### Risk of Hemorrhage

Major hemorrhage probability at 1 year was higher in the low-dose aspirin group than the control group (RR, 1.29 [95% CI, 1.05 to 1.53]; RD, 0.51% [95% CI, 0.13 to 0.91]) (Figure 2; eFigure 5 in Supplement 1). This difference was significant for digestive hemorrhage and other hemorrhages but not for intracranial hemorrhages (Table 2). NNH to provoke a major hemorrhage at 1 year was 196. Major hemorrhage risk was not increased at 3 years in the low-dose aspirin group.

### Net Clinical Benefit

Net clinical benefit was not different across groups at 1 year. There was also no significant difference at 3 years (RR, 0.93 [95% CI, 0.86 to 1.00]; RD, −0.80% [95% CI, −1.64% to 0.01%).

### Per-Protocol Analysis

In the observational per-protocol analysis, the reduction in MACE risk associated with low-dose aspirin was similar to that observed in the ITT analogue analysis (1-year RD, −0.77% [95% CI, −1.24 to −0.31]; 3-year RD, −1.74% [95% CI, −2.69 to −0.80]). The 1-year risk of major hemorrhage was also similar to ITT findings (RD, 0.41% [95% CI, 0.01 to 0.81]) (eFigure 6 and eTable 9 in Supplement 1).

### Subgroup Analyses

**Figure 3.**
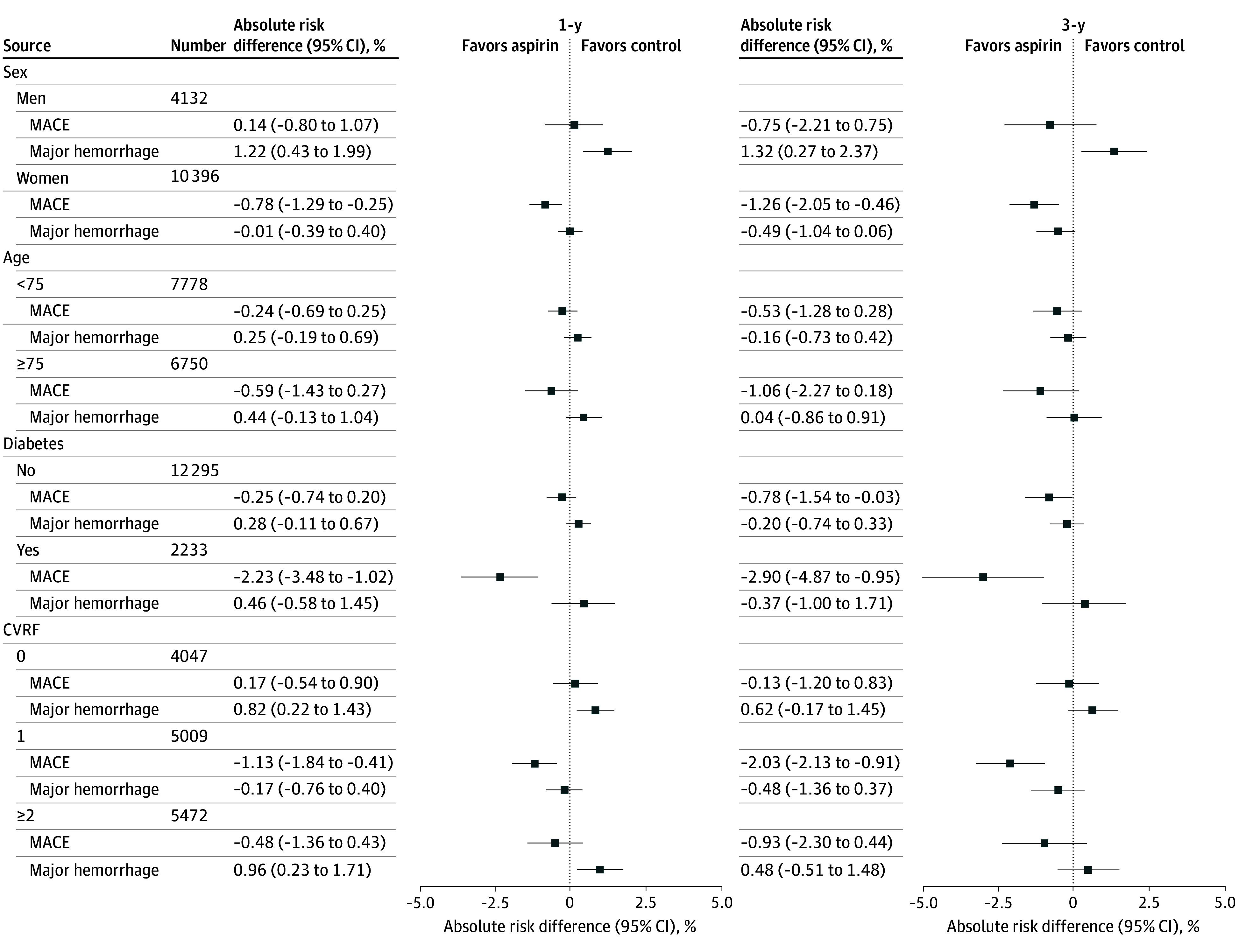
Dot Plots of Difference in Probability of Major Cardiovascular and Hemorrhagic Events in Different Subgroups CVRF indicates cardiovascular risk factors; MACE, major cardiovascular (composite outcome of all-cause mortality, myocardial infarction, and ischemic stroke). Major hemorrhage is a composite event of intracranial bleeding, digestive bleeding, and hospitalization for other hemorrhage. Risk difference is expressed as the difference in probability between the low-dose aspirin group and the control group.

Subgroup analyses for MACE and major hemorrhage at 1 year and 3 years are presented in Figure 3 and eFigure 7, eTable 10, and eTable 11 in Supplement 1. A clear difference in pattern was observed between men and women. Among men, at 1 year, we observed a higher risk of hemorrhage in the low-dose aspirin group (RD, 1.22% [95% CI, 0.43 to 1.99]) without any reduction in the risk of MACE (RD, 0.14% [95% CI, −0.80% to 1.07%]). In contrast, we observed an association between low-dose aspirin and reduced risk of MACE in women (1-year RD, −0.78% [95% CI, −1.29% to −0.25%]) without increase in risk of major hemorrhage (1-year RD, 0.01% [95% CI, −0.39% to 0.40%]) despite similar baseline characteristics between men and women (eTable 12 in Supplement 1). Patients with diabetes at baseline showed the largest association between low-dose aspirin use and lower MACE risk (1-year RD, −2.23% [95% CI, −3.48% to −1.02%]).

### Sensitivity Analyses

Results were generally similar with a more pronounced net clinical benefit association in favor of low-dose aspirin when excluding patients with any history of low-dose aspirin prescription (eFigure 9 in Supplement 1); however, there was a slight increase in major hemorrhage. After excluding patients with prevalent corticosteroid, methotrexate, or tocilizumab use, major hemorrhages were more frequent in the low-dose aspirin group, while MACE and all-cause mortality did not differ significantly between groups (eFigure 10 in Supplement 1). Censoring patients without corticosteroid dispensed by day 30, shortening the grace period to 7 days, or restricting inclusions from January 1, 2016, onward showed similar results (eFigures 10-13 in Supplement 1), as did applying a 1-month carry-over period on low-dose aspirin for bleeding events.

### Analyses With Control Outcome and Control Exposure

As expected, the risks of infection at 1 and 3 years were not different according to low-dose aspirin exposure (eFigure 14 in Supplement 1). Consistent with other studies, individuals exposed to laxatives were at higher risk of MACE and all-cause mortality^26,27^ (eFigure 15 in Supplement 1). However, as anticipated, the incidence of major hemorrhage did not differ between groups and showed no variation by sex (eFigure 16 in Supplement 1).

## Discussion

In this retrospective cohort study, we emulated a target trial to assess the association of low-dose aspirin initiation at GCA diagnosis with risk of MACE in patients without prior CVD. We first found that approximately one-third of patients without history of CVD received low-dose aspirin at GCA diagnosis in France, with no clear association with age, sex, or CVD risk profile. At 1 year, low-dose aspirin was associated with a reduction in MACE, including all-cause mortality. However, low-dose aspirin was also associated with an increased risk of major hemorrhage. At 3 years, low-dose aspirin was associated with reduced risk of MACE, including myocardial infarction and stroke, with no association with risk of major hemorrhage. The association between low-dose aspirin and reduced MACE risk was more pronounced in women and in patients with diabetes.

Three recent randomized trials have evaluated aspirin for primary CVD prevention in adults without GCA. The ASPREE trial,^10^ conducted in healthy individuals with a mean age similar to that of our study population, found no benefit on MACE over 6 years and an increased risk of hemorrhage, with a low incidence of MACE overall. The ASCEND trial,^28^ limited to patients with diabetes aged older than 40 years, showed an overall benefit of low-dose aspirin on MACE, especially during the first 3 years but with a significantly increased risk of bleeding. Finally, in the ARRIVE trial,^11^ which excluded patients at high risk of bleeding and those with diabetes, low-dose aspirin did not reduce MACE risk in the ITT analysis but increased the risk of bleeding. In 2 subsequent meta-analyses,^29,30^ low-dose aspirin was associated with a lower risk of myocardial infarction but no significant difference was found for all-cause mortality.

These conflicting findings suggest that the potential benefit of low-dose aspirin may be population-specific, underscoring the importance of identifying subgroups in whom benefits outweigh risks.^31^ Accordingly, some experts have advocated for a more parsimonious and highly selective use of low-dose aspirin in primary prevention.^32^ These discrepancies directly motivated our study. Patients with chronic inflammatory diseases are at elevated cardiovascular risk, with patients with GCA appearing particularly vulnerable,^1,2^ possibly due to a higher prevalence of baseline risk factors^33^ and prolonged corticosteroid exposure.

We observed a lower risk of myocardial infarction at 3 years associated with low-dose aspirin, consistent with a 2019 meta-analysis.^30^ In contrast, the observed reduction in stroke is not consistent with findings from a 2020 meta-analysis.^34^ Moreover, we observed a reduction in all-cause mortality, which differs from the results of the ASPREE trial.^35^ ASPREE unexpectedly showed a higher cancer-related mortality rate in the aspirin group, particularly after 4 years of follow-up. In our study, the difference in all-cause mortality emerged as early as 6 months. These apparently conflicting findings may reflect substantial differences in study populations (GCA patients or community-dwelling elderly) and indications for low-dose aspirin use across studies. Therefore, replication of our results is required to confirm our observations.

A notable finding was a sex-specific difference in the association between low-dose aspirin and MACE. Women exposed to low-dose aspirin had a lower 1-year risk of MACE without a significant increase in bleeding risk, whereas men experienced higher bleeding risk without an apparent cardiovascular benefit. This observation is noteworthy, given the persistent underrepresentation of women in cardiovascular trials.^36^ In addition, the association between low-dose aspirin and reduced MACE risk was stronger in patients with diabetes, consistent with the ASCEND trial.^28^

With respect to safety, a 2016 meta-analysis^37^ reported a higher bleeding risk in men than in women, in line with our results. In contrast, most randomized clinical trials of low-dose aspirin in primary prevention have not identified any sex-specific differences in cardiovascular outcomes,^10,11,28^ although these trials predominantly enrolled men. The Women’s Health Study, a randomized clinical trial conducted exclusively in women, did not demonstrate a benefit for its primary end point of MACE.

Taken together, our findings may reflect population-specific mechanisms, such as underdiagnosed CVD in women before GCA diagnosis, related to atypical symptoms and distinct plaque characteristics,^38^ potentially contributing to the early associations between low-dose aspirin use and reduced MACE risk in women. Alternatively, residual bias related to the observational design cannot be excluded, and replication in other populations with chronic inflammatory diseases is warranted.

### Limitations

This study has some limitations. First, residual confounding cannot be excluded, although negative outcome and control exposure analyses did not suggest a strong residual confounder. Second, we used all-cause rather than cardiovascular mortality, as causes of death derived from death certificates may be unreliable, particularly for sudden deaths outside health care facilities, and are not adjudicated by experts.^39^ Third, although *International Statistical Classification of Diseases and Related Health Problems, Tenth Revision* (*ICD-10*) codes for GCA have high specificity,^17^ some patients may have been included at relapse rather than at GCA onset, as suggested by frequent corticosteroid use in the year prior. These patients may represent prevalent polymyalgia rheumatica. However, our sensitivity analysis excluding these patients did not change the direction of the results, although statistical significance was affected. Moreover, because our study included all patients with a hospital diagnosis of GCA, generalizability may be limited. Fourth, proton pump inhibitors are widely prescribed in France, and more than half of the study population was exposed by the end of the grace period, which may have lowered baseline bleeding risk compared with other health care settings. Fifth, the low-dose aspirin group showed higher incidences of AOIN and lower-limb ischemia, possibly due to protopathic bias, although absolute numbers of cases were low. The SNDS may not fully capture clinical features prompting low-dose aspirin initiation, such as symptomatic lower-limb arterial stenosis or transient visual loss, which may have limited our ability to fully balance groups for individuals at very high underlying vascular risk. We believe the observation of a protective association with low-dose aspirin despite this limitation supports the robustness of the finding.

## Conclusions

In this retrospective cohort study, initiation of low-dose aspirin at the time of in-hospital diagnosis of GCA in patients without history of CVD was associated with a lower 1-year risk of MACE but a higher risk of major hemorrhage. At 3 years, low-dose aspirin was associated with a significant reduction in MACE risk without an increased risk of major hemorrhage. Subgroup analyses suggested greater benefit in women and in patients with diabetes at GCA diagnosis. These findings need confirmation and replication in patients with chronic inflammatory conditions.

## Notes

Supplement 1. eAppendix. Details on cloning censoring and weighting modelling eTable 1. Definition of the target trial and the emulated trial eTable 2. *ICD-10* codes, specific procedures codes and ATC codes eTable 3. Cox Model estimates for estimation of censoring weights during the grace period eTable 4. Cox Model estimates for estimation of censoring weights in second per-protocol definition eTable 5. Patients’ drug delivery, characteristics, missing variables and results after imputation eTable 6. Risk differences at multiple endpoints for secondary outcomes in pseudo-intention-to-treat eTable 7. Cumulative probability, risk difference and relative risk of primary and secondary outcomes at 6-month and 2-year in pseudo-intention-to-treat eTable 8. Restricted average causal effects at difference timepoints in pseudo-intention-to-treat analysis eTable 9. Cumulative probability, risk difference and relative risk of primary and secondary outcomes in per-protocol analysis eTable 10: Subgroup analyses for MACE and major hemorrhage at 1 year and 3 years eTable 11. Risk difference and relative risk of primary and secondary outcomes at 1 year according to sex eTable 12. Patients’ characteristics of patients according to sex eFigure 1. Flow chart eFigure 2. Standardized differences across treatment arms by the end of the grace period, before and after inverse probability of censoring weighting eFigure 3. Proportion of patients initiating low-dose aspirin by year of GCA diagnosis eFigure 4. Probability of deviating from assigned treatment arm after the grace period eFigure 5. Cumulative incidence of secondary cardiovascular and hemorrhagic endpoints eFigure 6. Cumulative incidence of main and secondary endpoints and risk difference at pre-defined timepoints in the per-protocol analysis eFigure 7. Probability of MACE and major hemorrhage according to sex and age at Giant Cell Arteritis diagnosis eFigure 8. Cumulative incidence of main and secondary endpoints according to sex eFigure 9. Cumulative incidence of main and secondary endpoints and risk difference at pre-defined timepoints after exclusion of patients with any history of low-dose aspirin deliverance eFigure 10. Cumulative incidence of main and secondary endpoints and risk difference at pre-defined timepoint after exclusion of patients with frequent steroid use or methotrexate or tocilizumab at baseline (sensitivity) eFigure 11. Cumulative incidence of main and secondary endpoints and risk difference at pre-defined timepoint after censoring patients without any steroid delivery at 30-day following index date eFigure 12. Cumulative incidence of main and secondary endpoints and risk difference at pre-defined timepoint with grace period of 7 days (sensitivity) eFigure 13. Cumulative incidence of main and secondary endpoints and risk difference at pre-defined timepoint within inclusions starting January 1, 2016 eFigure 14. Cumulative incidence of infection and composite outcome of infection or death and risk difference at pre-defined timepoints eFigure 15. Cumulative incidence of main and secondary endpoints and risk difference at pre-defined timepoint according to laxative delivery during grace period eFigure 16. Difference in MACE and major hemorrhage in different subgroups according to laxative delivery during grace period

Supplement 2. Data Sharing Statement
