# Supplementary material for: Low-Dose Aspirin for Cardiovascular Disease Primary Prevention in Patients With Giant Cell Arteritis
Source: JAMA Netw Open. 2026 Apr 17;9(4):e266579. doi: 10.1001/jamanetworkopen.2026.6579 (PMC13090850; doi:10.1001/jamanetworkopen.2026.6579)
Supplement: Supplement 1. — eAppendix. Details on cloning censoring and weighting modelling eTable 1. Definition of the target trial and the emulated trial eTable 2. ICD-10 codes, specific procedures codes and ATC codes eTable 3. Cox Model estimates for estimation of censoring weights during the grace period eTable 4. Cox Model estimates for estimation of censoring weights in second per-protocol definition eTable 5. Patients’ drug delivery, characteristics, missing variables and results after imputation eTable 6. Risk differences at multiple endpoints for secondary outcomes in pseudo-intention-to-treat eTable 7. Cumulative probability, risk difference and relative risk of primary and secondary outcomes at 6-month and 2-year in pseudo-intention-to-treat eTable 8. Restricted average causal effects at difference timepoints in pseudo-intention-to-treat analysis eTable 9. Cumulative probability, risk difference and relative risk of primary and secondary outcomes in per-protocol analysis eTable 10: Subgroup analyses for MACE and major hemorrhage at 1 year and 3 years eTable 11. Risk difference and relative risk of primary and secondary outcomes at 1 year according to sex eTable 12. Patients’ characteristics of patients according to sex eFigure 1. Flow chart eFigure 2. Standardized differences across treatment arms by the end of the grace period, before and after inverse probability of censoring weighting eFigure 3. Proportion of patients initiating low-dose aspirin by year of GCA diagnosis eFigure 4. Probability of deviating from assigned treatment arm after the grace period eFigure 5. Cumulative incidence of secondary cardiovascular and hemorrhagic endpoints eFigure 6. Cumulative incidence of main and secondary endpoints and risk difference at pre-defined timepoints in the per-protocol analysis eFigure 7. Probability of MACE and major hemorrhage according to sex and age at Giant Cell Arteritis diagnosis eFigure 8. Cumulative incidence of main and secondary endpoints according to sex eFigure 9. Cumu [file jamanetwopen-e266579-s001.pdf]

Supplemental Online Content

Beydon M, Hajage D, Guedon AF, Carrat F, Seror R, Tubach F. Low-dose aspirin for cardiovascular disease primary prevention in patients with giant cell arteritis. *JAMA Netw Open*. 2026;9(4):e266579. doi:10.1001/jamanetworkopen.2026.6579

eAppendix. Details on cloning censoring and weighting modelling

eTable 1. Definition of the target trial and the emulated trial

eTable 2. ICD-10 codes, specific procedures codes and ATC codes

eTable 3. Cox Model estimates for estimation of censoring weights during the grace period

eTable 4. Cox Model estimates for estimation of censoring weights in second per-protocol definition

eTable 5. Patients' drug delivery, characteristics, missing variables and results after imputation

eTable 6. Risk differences at multiple endpoints for secondary outcomes in pseudo-intention-to-treat

eTable 7. Cumulative probability, risk difference and relative risk of primary and secondary outcomes at 6-month and 2-year in pseudo-intention-to-treat

eTable 8. Restricted average causal effects at difference timepoints in pseudo-intention-to-treat analysis

eTable 9. Cumulative probability, risk difference and relative risk of primary and secondary outcomes in per-protocol analysis

eTable 10 : Subgroup analyses for MACE and major hemorrhage at 1 year and 3 years

eTable 11. Risk difference and relative risk of primary and secondary outcomes at 1 year according to sex

eTable 12. Patients' characteristics of patients according to sex

eFigure 1. Flow chart

eFigure 2. Standardized differences across treatment arms by the end of the grace period, before and after inverse probability of censoring weighting

eFigure 3. Proportion of patients initiating low-dose aspirin by year of GCA diagnosis

eFigure 4. Probability of deviating from assigned treatment arm after the grace period

eFigure 5. Cumulative incidence of secondary cardiovascular and hemorrhagic endpoints

eFigure 6. Cumulative incidence of main and secondary endpoints and risk difference at pre-defined timepoints in the per-protocol analysis

© 2026 Beydon M et al. *JAMA Network Open*.

eFigure 7. Probability of MACE and major hemorrhage according to sex and age at Giant Cell Arteritis diagnosis

eFigure 8. Cumulative incidence of main and secondary endpoints according to sex

eFigure 9. Cumulative incidence of main and secondary endpoints and risk difference at pre-defined timepoints after exclusion of patients with any history of low-dose aspirin deliverance

eFigure 10. Cumulative incidence of main and secondary endpoints and risk difference at pre-defined timepoint after exclusion of patients with frequent steroid use or methotrexate or tocilizumab at baseline (sensitivity)

eFigure 11. Cumulative incidence of main and secondary endpoints and risk difference at pre-defined timepoint after censoring patients without any steroid delivery at 30-day following index date

eFigure 12. Cumulative incidence of main and secondary endpoints and risk difference at pre-defined timepoint with grace period of 7 days (sensitivity)

eFigure 13. Cumulative incidence of main and secondary endpoints and risk difference at pre-defined timepoint within inclusions starting January 1, 2016

eFigure 14. Cumulative incidence of infection and composite outcome of infection or death and risk difference at pre-defined timepoints

eFigure 15. Cumulative incidence of main and secondary endpoints and risk difference at pre-defined timepoint according to laxative delivery during grace period

eFigure 16. Difference in MACE and major hemorrhage in different subgroups according to laxative delivery during grace period

This supplemental material has been provided by the authors to give readers additional information about their work.

© 2026 Beydon M et al. *JAMA Network Open*.

Details on cloning censoring and weighting modelling

In order to ensure all patients shared the same time of inclusion, treatment allocation and follow-up start, we applied the cloning, censoring and weighting approach. At hospital discharge, all observations were copied in both treatment allocation arms: ASA initiation within 14-days arm (further referred to as ASA arm) and no ASA initiation within 14-days arm (further referred to as control arm). At this starting point, we doubled the number of individual observations.

During this 14-day grace period, copies in each arm were censored whenever they deviate from their assigned strategy: delivery of ASA in the control arm or no delivery in the aspirin arm. Any event occurring during this grace period, was affected to all uncensored clones. By day 14, the uncensored population included the number of individuals at baseline except for individuals who experienced an event during the 14-day period.

Since censoring is informative, censoring probability needs to be balanced across treatment arms using inverse probability of censoring weights. A pseudo-population of upweighted uncensored observations is created with weights defined as:

$$W_g = \prod_{k=0}^{13} \frac{1}{\Pr\left(C_k^g \mid C_{k-1}^g = 0, A_{k-1}, L_{k-1}\right)}$$

With k an indicator of time in days during the grace period, C^g censorship during the grace period, A allocation arm and L a vector of time-varying covariates evaluated at baseline and throughout the grace period. Weights can be estimated using a Cox model with censoring as outcome variable and treatment arm as well as time-dependent covariates as independent variables.

In the pseudo-ITT analysis, weights are not further modified after day 14. In the per-protocol analysis, individuals in either arm can further be censored in case of deviation from treatment allocation arm: if ASA was introduced in the control arm (unless otherwise indicated by a CV event) or interrupted for more than 90 days in the aspirin arm (unless due to a major hemorrhage), or in case of deliverance of another antiplatelet or anticoagulant in either arm.

This censoring is also informative and therefore further IPCW is needed using the same principal:

$$W_f = \prod_{k=14}^{K} \frac{1}{\Pr\left(C_k^f \mid C_{k-1}^f = 0, A_{k-1}, L_{k-1}\right)}$$

With C^f censorship during follow-up after the initial grace period. With the final weights are therefore defined as:

$$W = W_g \times W_f = \prod_{k=0}^{13} \frac{1}{\Pr\left(C_k^g \mid C_{k-1}^g = 0, A_{k-1}, L_{k-1}\right)} \times \prod_{k=14}^{K} \frac{1}{\Pr\left(C_k^f \mid C_{k-1}^f = 0, A_{k-1}, L_{k-1}\right)}$$

In this study, the set of covariates of the two models were almost identical, except that the second model of the per-protocol analysis included cumulative steroid consumption. In the second model, censor status and covariates were updated by 30-days intervals for computational efficiency. Few missing variables were observed for socio-economic variables, and were imputed using multiple chained equations (eTable 5).

Control exposure and outcome

In order to explore residual confounding, we set two types of controls, one regarding outcome and one regarding exposure.

The outcome negative control was defined as incidence of infection, defined as hospitalization with a main diagnosis of infection. No association between ASA dispensation at GCA diagnosis and infection risk was expected, therefore, any observed association would suggest the presence of residual confounding. All ICD-10 codes are listed in eTable 2. We adjusted also on the number of hospitalizations for infection in the 3 years preceding diagnosis. Results of this analysis are shown in eFigure 14.

To further assess the robustness of our findings regarding the principal outcomes and in particular hemorrhagic risk, we defined a control exposure. We considered laxatives as the control intervention, as their prescription is expected to be driven primarily by patient characteristics rather than physician adherence to specific guidelines.

Use of laxatives and constipation have been shown to be associated with all-cause mortality and MACE in cohort studies (Salmoirago-Blotcher 2011, Sumida 2019, Kubota 2016) with an HR of 1.12 (95% CI 1.11 to 1.13) for all-cause mortality, 1.19 (1.15 to 1.22) for coronary disease and 1.19 (1.14 to 1.23) for stroke risk (Sumida 2019). In our population, we therefore expected to observe an increase in MACE and mortality among patients with laxative delivery at GCA baseline without increase in major hemorrhage.

The control intervention was introduction of laxatives at GCA diagnosis (ATC codes eTable 2). We defined a 14 days grace period from index date during which individuals were cloned in the laxative and no laxative group, and further censored if they deviated from allocation arm. The evaluated outcomes were MACE, major hemorrhage, all-cause mortality and net clinical benefit. Adjustment variables were identical to the main analysis. We further explored MACE and hemorrhage risk according to age, sex and number of cardiovascular risk factors. Results are shown in eFigure 15-16.

eTable 1: Definition of the Target Trial and the Emulated Trial

	Target Trial	Emulated Trial
**Eligibility Criteria**	- Incident hospital diagnosis of GCA<br>- Age ≥ 50 years at the time of GCA diagnosis<br>- Alive at hospital discharge<br>- Hospitalization duration of less than 28 days<br>- Community-dwelling (i.e., exclusion of nursing home residents)<br>- No history of cardiovascular events (myocardial infarction, stable or unstable angina, ischemic stroke, significant cerebral artery stenosis ≥ 70%, symptomatic peripheral artery disease (PAD)), nor synchronous CV event at diagnosis<br>- No treatment with antiplatelet or anticoagulant therapy within 3 months of GCA diagnosis, no history of atrial fibrillation<br>- No diagnosis of acute ischemic optic neuropathy prior to or synchronous with GCA diagnosis	Same as target trial except:
**Intervention**	Introduction of low dose of aspirin (ASA) at a dose of 75 to 300 mg/day vs.<br>No introduction of ASA at hospital discharge	Same as target trial
**Assignment to Intervention**	Patients are assigned at Day 0 by randomization to one of the two compared interventions: ASA or no ASA.	Patients are assigned at Day 0 (GCA diagnosis hospital discharge) to both interventions.<br><br>Each clone is censored during or at the end of the 14-day grace period as soon as it deviates from the intervention of the assigned group.
**Outcomes**	**Primary outcome at 1 year**<br>Major cardiovascular event, composite endpoint, defined as:<br>- Acute myocardial infarction<br>- Ischemic stroke (transient or constituted)<br>- All-cause death<br><br>**Secondary outcomes**<br>- Primary endpoint at 6 months, 2 years, and 3 years<br>- Each component of the primary endpoint (Stroke transient or established, acute myocardial infarction, all-cause death)<br>- Occurrence of major hemorrhage (intracranial hemorrhage, hospitalization for gastrointestinal bleeding, other bleeding leading to a hospitalization)<br>- Net clinical benefit: Composite of primary endpoint and major hemorrhage<br>- Cardiovascular death	Same as target trial

© 2026 Beydon M et al. *JAMA Network Open*.

	Target Trial	Emulated Trial
	- Acute ischemia or revascularization of lower limbs<br>- Coronary events defined as acute myocardial infarction or coronary revascularization<br>- Cardiovascular events defined as a composite: Acute myocardial infarction or coronary revascularization, ischemic stroke (transient or established), acute ischemia, revascularization or limb amputation, cardiovascular death<br>- AOIN	
**Follow-up**	From randomization until the event, loss to follow-up, or the end of the 3-year follow-up period. All patients were followed at least 1 year	From eligibility and cloning (Day 0) until the event or the end of the study follow-up period (3 years), or the end of the observation period (December 31, 2023).<br>All patients were followed at least 1 year.
**Causal Contrast**	ITT (Intention-to-Treat) and per-protocol	Observational analog of ITT (Pseudo-ITT: patients are followed according to their treatment arm by the end of the grace period)<br><br>Per-protocol: patients are censored in case of introduction of aspirin in the control arm (unless introduction indicated by ischemic event) or aspirin interruption in the aspirin arm (unless interruption indicated by a hemorrhagic event), or introduction of antiplatelet or anticoagulant in any arm
**Statistical Analysis**	ITT: between-group risk difference at 1 year difference tested using a Kaplan-Meier estimator<br><br>Per-protocol: Between-group risk difference using weighted Kaplan-Meier estimator weighted by the probability of censoring in case of protocol deviation	Pseudo-ITT: between-group risk difference at 1 year difference tested using a Kaplan-Meier estimator<br><br>Per-protocol: Between-group risk difference using weighted Kaplan-Meier estimator weighted by the probability of censoring in case of protocol deviation

© 2026 Beydon M et al. *JAMA Network Open*.

eTable 2: ICD-10 codes, specific procedures codes and ATC codes

	ICD-10 codes	Specific procedures (CCAM codes)	ATC codes	Definition
**Diseases**				
Giant cell arteritis	M315, M316			
Polymyalgia rheumatica	M353, M315			
**Antiplatelet**				
Low-dose Aspirin			B01AC06<br>C10BX02	Any pharmacy delivery. Covered period by a delivery depends on number of tablets per dispensation unit.
Clopidogrel			B01AC04	
Prasugrel			B01AC22	
Ticargrelor			B01AC24	
**Anticoagulant**				
Coumadin			B01AA03	Any pharmacy delivery. Covered period by a delivery depends on number of tablets per dispensation unit.
Fluindion			B01AA12	
Enoxaparin			B01AB05	
Tinazaparin			B01AB10	
Calciparin			B01AB01	
Fondaparinux			B01AB01	
Apixaban			B01AF02	
Rivaroxaban			B01AF01	
**Covariates**				
Diabetes	E10, E11, E12, E13, E14, G590, G632, G730, G990, H280, H360, I792, L97, M142, M146, N083		A10* except A10BK01, A10BK02, A10BK03, A10BK04, A10BX06	
Hypertension	H350, I10, I11, I12, I13, I110, I119, I120, I129, I130, I131, I132, I139,		C02AC01, C02CA01, C02DC01, C02LA01, C02AB02, C02AC02, C02AC05, C02AC06, C02CA06, C03AA01, C03CA01, C03DA01,	

© 2026 Beydon M et al. *JAMA Network Open*.

	I150, I151, I152, I158, I159	C03DB01, C03EA01, C03CA02, C03AA03, C03BX03, C03CA03, C03BA04, C03EA04, C03BA10, C03BA11, C07AG01, C07AA02, C07AB02, C07BA02, C07BB02, C07FB02, C07AA03, C07AB03, C07BB03, C07CA03, C07FB03, C07AB04, C07AA05, C07AB05, C07AA06, C07DA06, C07AB07, C07BB07, C07AB08, C07AA12, C07AB12, C07BB12, C07AA15, C07AA16, C07AA23, C08CA01, C08CX01, C08DA01, C08DB01, C08CA02, C08GA02, C08CA03, C08CA04, C08CA05, C08CA08, C08CA09, C08CA11, C08CA13, C09AA01, C09BA01, C09CA01, C09DA01, C09DB01, C09AA02, C09BA02, C09BB02, C09BX02, C09CA02, C09DA02, C09DB02, C09XA02, C09AA03, C09BA03, C09CA03, C09DA03, C09AA04, C09BA04, C09BB04, C09CA04, C09DA04, C09DB04, C09AA05, C09BA05, C09AA06, C09BA06, C09CA06, C09DA06, C09AA07, C09BA07, C09BB07, C09CA07, C09DA07, C09AA08, C09CA08, C09DA08, C09AA09, C09BA09, C09AA10, C09BB10, C09AA13, C09AA15, C09BA15, C09AA16, C09XA52, C10BX03	Any ICD-10 code as long-term chronic disease or hospitalization discharge code (main or associate) or any delivery of treatment with compatible ATC code
Smoking	F17, J42, J43, J44, J430, J431, J432, J438, J439, J440,	R03AC18, R03AC19, R03BB04, R03BB05, R03BB06, R03BB07,	

© 2026 Beydon M et al. *JAMA Network Open*.

	J441, J448, J449, J961, Z716, Z720		R03AL04, R03AL05, R03AK04, N07BA01, N07BA02, N07BA03	
Dyslipidemia	E78		C10*	
Advanced chronic kidney disease	I120, N18			
Dementia	F00, F01, F03, F03, G30, G31			
Alcohol abuse	E512, F10, G312, G621, I426, K292, K70, K852, K860, R780, T510, T518, T519, X450, X65, Y15, Y90, Y91, Z502, Z714, Z721		N07BB01, N07BB03, N07BB04, N07BB05	
Atrial fibrillation	I48			
Cardiac insufficiency	I50, I110, I130, I50, J81		C09DX04	
Obesity	E66			
Falls	W00-W19			
Trauma	S00-S99			
Iron deficiency	D50		B03AA, B03AB, B03AC, B03AD, B03AE	
Steroid			H02AB06, H02AB07, H02AB04	Any pharmacy or in-hospital delivery
Tocilizumab			L04AC07	
Methotrexate			LA1BA01, L04AX03	
**Exclusion criteria**				
Ischemic heart disease	I20, I21, I22, I23, I24, I25	DDAA002, DDAF001, DDAF003, DDAF004, DDAF006, DDAF007, DDAF008, DDAF009, DDAF010, DDFF001, DDFF002, DDLF001, DDPF002, DDSF001, YYYY082, DDMA003, DDMA004, DDMA005, DDMA006, DDMA007,		Any ICD-10 code as long-term chronic disease or hospitalization discharge code (main or associate) or any in-hospital specific procedure codes

© 2026 Beydon M et al. *JAMA Network Open*.

		DDMA008, DDMA009, DDMA011, DDMA012, DDMA013, DDMA015, DDMA016, DDMA017, DDMA018, DDMA019, DDMA020, DDMA021, DDMA022, DDMA024, DDMA025, DDMA026, DDMA027, DDMA028, DDMA029, DDMA030, DDMA031, DDMA032, DDMA033, DDMA034, DDMA035, DDMA036, DDMA037, DDMA038, ENFA003, EPFA006
Ischemic cerebrovascular disease or carotid stenosis	G45, G450, G451, G452, G458, G459, I63, I64, I693, I694, I698, I65, I66	EAJF341, EAFA001, EANF002, EBNF001, EBNF002
Peripheral artery disease	I74, I702, I792	EDAF002, EDAF003, DGPF001, DGPF002, EDPF008, EDPF009, EDPF006, EDLF005, EDLF004, EDAF004, EDAF006, EDPF007, EDPF001, EDLF007, EEAF003, EEAF004, EEAF005, EEAF002, EEAF001, EEAF006, EENF002, EENF001, EEPF002, EEPF001, EEJF001, ENAF002, ENAF001, ENNF001, ENFF001, DGFA010, DGFA004, DGFA005,

© 2026 Beydon M et al. *JAMA Network Open*.

		DGFA012, DGFA011, DGFA001, DGFA009, DGFA003, DGFA007, DGFA008, EDFA006, EDFA003, EDFA007, DGCA012, DGCA007, DGCA019, DGCA009, DGCA026, DGCA022, DGCA010, DGCA004, DGCA030, DGCA020, DGCA029, EDCA003, DGFA015, DGKA004, EDEA001, EEFA004, EEFA002, EEFA001, EEFA003, EECA007, EECA006, EDCA005, EDCA004, EECA002, EECA001, EECA003, EECA008, EECA010, EECA005, EECA012, EEGA002, EEAA002, EEFA006, EEKA001, ENFA006, ENFA001, ENFA004, ENFA005	
Anterior ischemic optic neuropathy	H47, G453		
**Events**			
Acute myocardial infarction	I20, I21		Any ICD-10 code as long-term chronic disease or hospitalization discharge code (main only) or any in-hospital specific procedure codes
Transient or ischemic stroke	G45, I63, I64	EAJF341, EAFA001, EANF002, EBNF001, EBNF002	
Acute myocardial infarction or coronary revascularization	I20, I21	DDAA002, DDAF001, DDAF003, DDAF004, DDAF006, DDAF007, DDAF008, DDAF009, DDAF010, DDFF001, DDFF002, DDLF001, DDPF002, DDSF001,	

© 2026 Beydon M et al. *JAMA Network Open*.

		YYYY082, DDMA003, DDMA004, DDMA005, DDMA006, DDMA007, DDMA008, DDMA009, DDMA011, DDMA012, DDMA013, DDMA015, DDMA016, DDMA017, DDMA018, DDMA019, DDMA020, DDMA021, DDMA022, DDMA024, DDMA025, DDMA026, DDMA027, DDMA028, DDMA029, DDMA030, DDMA031, DDMA032, DDMA033, DDMA034, DDMA035, DDMA036, DDMA037, DDMA038, ENFA003, EPFA006	
Intracranial bleeding	I60, I61, I62, S063, S064, S065, S066, S068	HESE001, HESE002	
Gastro-intestinal bleeding	K250, K252, K260, K262, K270, K272, K280, K282, K290, K625, K920, K921, K922		
Other major hemorrhage	R042, N02, R040, J942, D62, H356, H431, H450		
Cardiovascular death (Available until December 31st 202)	I20, I21, I22, I23, I24, I25, I48, I49, I50, I63, I64, I74		
**Sensitivity analyses**			
Laxatives (controls exposure)			A06*
Infections	Cutaneous: J34, L01-L04, L050, L08, A46, M726, L88		

© 2026 Beydon M et al. *JAMA Network Open*.

	<u>Digestive:</u><br>K803, K830, K35, K800, K804, K810, K871, K8570, K572, K574, K578, A00-A09, K61, K630, K750, K770, K733, B15, B16, B17, B19, B251, K65<br><br><u>ENT:</u><br>H60, H62, H651, H651, H660, H664, H670, H671, K113, K122, K140, J01-J06, J36, J390, J391, H700, H680<br><br><u>Musculoskeletal:</u><br>M00, M01, M600, M630, M631, M632, M463, M465, M490, M491, M493, M864, M869, M900, M901, M902, M650<br><br><u>Urinary tract:</u><br>N700, N730, N733, N74, N751, N764, N410, N412, N413, N45, N482, N499, N080, N151, N300, N303, N308, N330, N340, N390, N10<br><br><u>Central nervous system:</u>	

© 2026 Beydon M et al. *JAMA Network Open*.

	G00-G02, G06, G07, G042, G041, A80-A89, A17<br><br><u>Respiratory tract:</u><br>J09-J17, J181, J188, J189, J20, J22, J65, J85, J86, A15, A16, A19, U049, U071<br><br><u>Other or septicemia:</u><br>A18, A20, A2*, A3*, A4*, A7*, B95, B96, I301, I320, I321, I33, I440, I410, I412, I430, H440, H441, B39, B44, B45, B5, B6, B7, B80, B81, B82, B83, A9*, B34, B97, E060, E236, E321, N61, O85, O86, R572, T814, T802	

© 2026 Beydon M et al. *JAMA Network Open*.

eTable 3: Cox Model estimates for estimation of censoring weights during the grace period (model from diagnosis date to end of grace period)

	Aspirin			Control		
	**Estimate**	**SE**	**p**	**Estimate**	**SE**	**p**
Sex (Female)	-0,484	0,198	0,01	0,716	0,259	0,006
Age at diagnosis	-0,011	0,005	0,02	0,016	0,006	0,008
Polymyalgia rheumatica ICD code	0,08	0,025	0,001	-0,137	0,035	<0,001
Length of hospital stay at diagnosis (days)	-0,013	0,002	<0,001	0,017	0,002	<0,001
Diabetes	-0,086	0,031	0,005	0,112	0,039	0,004
Hypertension	-0,103	0,023	<0,001	0,174	0,031	<0,001
Dyslipidemia	-0,059	0,023	0,010	0,086	0,031	0,005
Tobacco use	-0,068	0,034	0,048	0,107	0,044	0,014
Obesity	0,011	0,055	0,844	0,01	0,072	0,891
Cardiac insufficiency	-0,057	0,103	0,575	0,073	0,141	0,606
Cancer	0,048	0,03	0,111	-0,052	0,041	0,208
Dementia	0,164	0,057	0,004	-0,237	0,086	0,006
Chronic kidney disease	0,097	0,066	0,142	-0,128	0,097	0,186
Alcohol abuse	-0,049	0,078	0,532	0,076	0,097	0,437
Fracture or injury	-0,03	0,039	0,450	0,049	0,052	0,341
Falls	0,16	0,079	0,043	-0,246	0,117	0,035
Frequent corticosteroid use	0,194	0,027	<0,001	-0,311	0,041	<0,001
Polymedication	0,181	0,026	<0,001	-0,283	0,036	<0,001
Proton pomp inhibitor at baseline	-0,388	0,021	<0,001	0,79	0,03	<0,001
History of major hemorrhage	0,139	0,054	0,010	-0,248	0,078	0,001
Actual or past iron deficiency	0,059	0,026	0,025	-0,105	0,036	0,003
Tocilizumab	-0,007	0,091	0,941	-0,138	0,159	0,385
Methotrexate	0,001	0,059	0,984	0,118	0,085	0,163
General insurance scheme	0,024	0,031	0,430	-0,048	0,04	0,240
Deprivation index	-0,003	0,008	0,725	0,003	0,01	0,788
Full expanse coverage	0,037	0,051	0,471	-0,07	0,066	0,287
Year of diagnosis	-0,027	0,003	<0,001	0,038	0,004	<0,001
sex:AGE_ANN	0,007	0,003	0,008	-0,011	0,004	0,003

© 2026 Beydon M et al. *JAMA Network Open*.

eTable 4: Cox Model estimates for estimation of censoring weights in second per-protocol definition (model from end of grace period to end of follow-up)

	Aspirin			Control		
	**Estimate**	**SE**	**p**	**Estimate**	**SE**	**p**
Sex (Female)	0,136	0,345	0,693	-0,788	0,359	0,028
Age at diagnosis	0,006	0,008	0,479	-0,004	0,009	0,642
Polymyalgia rheumatica ICD code	0,044	0,044	0,312	-0,114	0,046	0,014
Length of hospital stay at diagnosis (days)	-0,018	0,004	0,000	0,007	0,003	0,032
Diabetes	-0,086	0,048	0,075	0,034	0,053	0,521
Hypertension	-0,02	0,04	0,616	0,165	0,044	<0,001
Dyslipidemia	-0,052	0,038	0,169	-0,003	0,041	0,935
Tobacco use	0,074	0,052	0,161	0,171	0,057	0,003
Obesity	-0,034	0,092	0,707	0,079	0,088	0,373
Cardiac insufficiency	0,536	0,153	<0,001	0,348	0,145	0,016
Cancer	0,078	0,05	0,120	0,217	0,05	<0,001
Dementia	0,103	0,096	0,284	-0,073	0,092	0,426
Chronic kidney disease	0,086	0,112	0,443	-0,021	0,108	0,844
Alcohol abuse	-0,003	0,118	0,980	0,033	0,128	0,797
Fracture or injury	0,099	0,059	0,096	0,086	0,063	0,170
Falls	0,114	0,124	0,359	0,109	0,117	0,355
Cumulative steroid (mg)	0	0	<0,001	0	0	0,003
Frequent corticosteroid use	0,014	0,051	0,783	-0,092	0,049	0,061
Polymedication	-0,113	0,045	0,013	0,052	0,045	0,252
Proton pomp inhibitor at baseline	-0,046	0,037	0,218	0,019	0,04	0,636
History of major hemorrhage	-0,145	0,107	0,174	-0,087	0,102	0,394
Actual or past iron deficiency	0,027	0,044	0,533	0,103	0,046	0,026
Tocilizumab	-0,014	0,095	0,880	-0,11	0,129	0,394
Methotrexate	-0,099	0,076	0,192	0,111	0,087	0,199
General insurance scheme	0,185	0,053	<0,001	-0,012	0,055	0,821
Deprivation index	-0,011	0,013	0,394	-0,014	0,014	0,321
Full expanse coverage	0,274	0,082	0,001	0,121	0,093	0,191
Year of diagnosis	-0,008	0,005	0,125	0,009	0,005	0,075
sex:AGE_ANN	-0,003	0,005	0,500	0,01	0,005	0,036

© 2026 Beydon M et al. *JAMA Network Open*.

eTable 5: Patients' drug delivery, characteristics, missing variables and results after imputation

	All patients N = 14,528	Aspirin N = 5,220	Control N = 9,269
Any delivery of steroids during follow-up	13,382 (92%)	4,954 (95%)	8,423 (91%)
Methotrexate delivery during follow-up	1,610 (11%)	569 (11%)	1,038 (11%)
Tocilizumab delivery during follow-up	855 (5.9%)	328 (6.3%)	525 (5.7%)
History of low-dose aspirin before 3-month prior to giant cell arteritis diagnosis	1,213 (8.3%)	451 (8.7%)	742 (8.1%)
Deprivation index (quantiles)			
1	2,810 (20%)	1,007 (20%)	1,775 (20%)
2	2,727 (19%)	968 (19%)	1,729 (19%)
3	2,878 (20%)	989 (20%)	1,862 (21%)
4	3,016 (21%)	1,093 (22%)	1,894 (21%)
5	2,685 (19%)	985 (20%)	1,661 (19%)
Missing	412	136	272
Deprivation index (quantiles) - imputed			
1	2,902 (20%)	1,035 (20%)	1,838 (20%)
2	2,779 (19%)	985 (19%)	1,763 (19%)
3	2,983 (21%)	1,022 (20%)	1,934 (21%)
4	3,102 (21%)	1,120 (22%)	1,953 (21%)
5	2,762 (19%)	1,016 (20%)	1,705 (19%)
Full expanse coverage	685 (4.7%)	245 (4.7%)	425 (4.6%)
Missing	8	0	8
Full expanse coverage - imputed	685 (4.7%)	245 (4.7%)	425 (4.6%)
General insurance scheme	12,493 (86%)	4,448 (86%)	7,906 (86%)

© 2026 Beydon M et al. *JAMA Network Open*.

eTable 6: Risk differences at multiple endpoints for secondary outcomes in pseudo-intention-to-treat

	1 year		3 years	
**Events**	**Risk difference (%) (CI)**	**Relative Risk (CI)**	**Risk difference (%) (CI)**	**Relative Risk (CI)**
Myocardial infarction or coronary revascularization	-0.13 (-0.34, 0.10)	0.90 (0.53, 1.24)	-0.65 (-1.02, -0.27)	0.72 (0.58, 0.87)
Cardiovascular death	-0.10 (-0.26, 0.06)	0.60 (0.26, 0.91)	-0.23 (-0.46, 0.02)	0.78 (0.56, 1.00)
Lower limb ischemia	0.13 (-0.06, 0.31)	1.71 (0.81, 2.48)	0.52 (0.24, 0.81)	1.65 (1.21, 2.07)
Anterior ischemic optic neuropathy	0.21 (0.05, 0.38)	2.14 (1.05, 3.12)	0.29 (0.11, 0.49)	1.73 (1.11, 2.33)
Cardiovascular events	-0.20 (-0.57, 0.19)	0.95 (0.77, 1.12)	-0.52 (-1.09, 0.08)	0.91 (0.81, 1.01)

Cardiovascular events is a composite outcome of myocardial infarction or coronary revascularization, cardiovascular death, stroke, and lower limb ischemia

© 2026 Beydon M et al. *JAMA Network Open*.

eTable 7: Cumulative probability, risk difference and relative risk of primary and secondary outcomes at 6-month and 2-year in pseudo-intention-to-treat

	6 months			2-year		
**Events**	**N events (%) ASA / Control**	**Absolute Difference (%) (CI)**	**Relative risk (CI)**	**N events (%) ASA / Control**	**Absolute Difference (%) (CI)**	**Relative risk (CI)**
**Primary outcome**	189 (2.14) / 297 (2.46)	-0.32 (-0.66, 0.01)	0.87 (0.74, 1.00)	386 (5.63) / 692 (6.31)	-0.69 (-1.27, -0.13)	0.89 (0.80, 0.98)
All-cause mortality	123 (1.16) / 221 (1.47)	-0.31 (-0.59, -0.05)	0.79 (0.61, 0.95)	268 (3.65) / 503 (4.04)	-0.39 (-0.86, 0.09)	0.90 (0.79, 1.02)
Myocardial infarction	29 (0.39) / 46 (0.41)	-0.02 (-0.18, 0.13)	0.95 (0.54, 1.33)	59 (0.91) / 121 (1.20)	-0.30 (-0.55, -0.04)	0.75 (0.56, 0.95)
Stroke	58 (0.65) / 71 (0.74)	-0.09 (-0.27, 0.10)	0.88 (0.63, 1.12)	90 (1.28) / 142 (1.50)	-0.22 (-0.50, 0.06)	0.85 (0.67, 1.03)
**Major hemorrhage**	95 (1.59) / 112 (1.12)	0.47 (0.16, 0.77)	1.42 (1.10, 1.73)	166 (3.08) / 276 (2.80)	0.28 (-0.17, 0.75)	1.10 (0.93, 1.27)
Digestive hemorrhage	55 (0.98) / 65 (0.67)	0.31 (0.06, 0.57)	1.47 (1.01, 1.91)	92 (1.82) / 139 (1.42)	0.40 (0.06, 0.75)	1.28 (1.01, 1.54)
Intracranial hemorrhage	28 (0.43) / 23 (0.22)	0.21 (0.06, 0.35)	1.93 (0.96, 2.78)	42 (0.65) / 61 (0.61)	0.04 (-0.16, 0.25)	1.07 (0.71, 1.41)
Other hemorrhage	39 (0.70) / 39 (0.38)	0.32 (0.12, 0.52)	1.83 (1.12, 2.47)	65 (1.27) / 98 (1.01)	0.27 (-0.02, 0.55)	1.27 (0.95, 1.57)
**Net clinical benefit**	249 (3.46) / 363 (3.31)	0.15 (-0.29, 0.59)	1.04 (0.91, 1.18)	497 (8.12) / 866 (8.39)	-0.28 (-0.98, 0.42)	0.97 (0.88, 1.05)

ASA: low-dose aspirin. Primary outcome is a composite outcome of all-cause mortality, myocardial infarction and stroke. Major hemorrhage is a composite of intracranial, digestive and other hemorrhage. Other hemorrhage is defined by a hospitalization with main diagnostic code for epistaxis, hematuria, hemothorax or intraocular hemorrhage.

Risk difference is given by the difference in probability in the low-dose aspirin arm and the control arm.

© 2026 Beydon M et al. *JAMA Network Open*.

eTable 8: Restricted average causal effects at difference timepoints in pseudo-intention-to-treat analysis

	Restricted average causal effect (Difference in restricted survival time in aspirin and control arm) (days) (CI)			
**Events**	**6 months**	**1 year**	**2 years**	**3 years**
**Primary outcome**	0.29 (-0.06, 0.66)	1.07 (0.09, 2.10)	3.08 (0.57, 5.77)	6.60 (2.21, 11.26)
All-cause mortality	0.25 (-0.03, 0.54)	0.90 (0.12, 1.70)	2.26 (0.16, 4.36)	4.05 (0.30, 7.79)
Myocardial infarction	-0.01 (-0.20, 0.17)	0.08 (-0.42, 0.57)	0.73 (-0.54, 1.97)	2.16 (-0.05, 4.29)
Stroke	0.12 (-0.08, 0.33)	0.37 (-0.18, 0.94)	1.06 (-0.33, 2.46)	2.15 (-0.22, 4.56)
**Major hemorrhage**	-0.48 (-0.82, -0.15)	-1.42 (-2.34, -0.52)	-2.68 (-5.05, -0.40)	-3.69 (-7.70, 0.18)
Digestive hemorrhage	-0.39 (-0.67, -0.11)	-1.15 (-1.91, -0.39)	-2.58 (-4.45, -0.69)	-3.67 (-6.76, -0.56)
Intracranial hemorrhage	-0.16 (-0.31, -0.00)	-0.39 (-0.81, 0.03)	-0.67 (-1.72, 0.38)	-1.20 (-2.99, 0.56)
Other hemorrhage	-0.34 (-0.56, -0.12)	-1.09 (-1.69, -0.50)	-2.29 (-3.76, -0.82)	-3.12 (-5.58, -0.64)
**Net clinical benefit**	-0.18 (-0.65, 0.30)	-0.47 (-1.79, 0.87)	-0.13 (-3.44, 3.29)	2.17 (-3.52, 8.02)

Primary outcome is a composite outcome of all-cause mortality, myocardial infarction and stroke. Major hemorrhage is a composite of intracranial, digestive and other hemorrhage. Other hemorrhage is defined by a hospitalization with main diagnostic code for epistaxis, hematuria, hemothorax or intraocular hemorrhage.

RACE is given by the difference in restricted mean survival time between aspirin arm and control

© 2026 Beydon M et al. *JAMA Network Open*.

eTable 9: Cumulative probability, risk difference and relative risk of primary and secondary outcomes in per-protocol analysis

	1-year			3-year		
Events	N events (%) ASA / Control	Absolute Difference (%) (CI)	Relative risk (CI)	N events (%) ASA / Control	Absolute Difference (%) (CI)	Relative risk (CI)
**Primary outcome**	208 (2.92) / 396 (3.68)	-0.77 (-1.24, -0.31)	0.79 (0.67, 0.91)	291 (6.83) / 703 (8.57)	-1.74 (-2.69, -0.80)	0.80 (0.69, 0.90)
All-cause mortality	137 (1.65) / 291 (2.22)	-0.57 (-0.93, -0.17)	0.75 (0.59, 0.90)	192 (4.38) / 487 (5.28)	-0.90 (-1.65, -0.12)	0.83 (0.69, 0.97)
Myocardial infarction	35 (0.58) / 60 (0.62)	-0.03 (-0.24, 0.17)	0.95 (0.60, 1.27)	49 (1.12) / 131 (1.84)	-0.72 (-1.13, -0.34)	0.61 (0.41, 0.79)
Stroke	58 (0.74) / 93 (1.05)	-0.31 (-0.54, -0.08)	0.71 (0.51, 0.90)	74 (1.57) / 154 (2.03)	-0.45 (-0.93, 0.02)	0.78 (0.55, 1.00)
**Major hemorrhage**	105 (2.08) / 151 (1.68)	0.41 (0.01, 0.81)	1.24 (0.98, 1.49)	135 (3.62) / 268 (3.46)	0.16 (-0.50, 0.87)	1.05 (0.85, 1.25)
Digestive hemorrhage	64 (1.40) / 81 (0.94)	0.45 (0.13, 0.78)	1.48 (1.07, 1.88)	76 (2.04) / 136 (1.76)	0.28 (-0.20, 0.76)	1.16 (0.87, 1.44)
Intracranial hemorrhage	24 (0.35) / 33 (0.38)	-0.02 (-0.18, 0.13)	0.94 (0.49, 1.35)	35 (0.76) / 56 (0.74)	0.03 (-0.28, 0.32)	1.03 (0.60, 1.44)
Other hemorrhage	42 (0.85) / 49 (0.50)	0.35 (0.11, 0.59)	1.71 (1.07, 2.29)	51 (1.38) / 98 (1.25)	0.13 (-0.29, 0.55)	1.10 (0.75, 1.45)
**Net clinical benefit**	278 (4.70) / 484 (4.89)	-0.19 (-0.79, 0.40)	0.96 (0.84, 1.08)	381 (9.60) / 873 (11.05)	-1.45 (-2.59, -0.37)	0.87 (0.77, 0.96)

ASA: low-dose aspirin. Primary outcome is a composite outcome of all-cause mortality, myocardial infarction and stroke. Major hemorrhage is a composite of intracranial, digestive and other hemorrhage. Other hemorrhage is defined by a hospitalization with main diagnostic code for epistaxis, hematuria, hemothorax or intraocular hemorrhage.

Risk difference is given by the difference in probability in the low-dose aspirin arm and the control arm.

© 2026 Beydon M et al. *JAMA Network Open*.

eTable 10: Subgroup analyses for MACE and major hemorrhage at 1 year and 3 years

		1-year risk difference % (95 CI)		3-year risk difference % (95 CI)	
		**MACE**	**Major hemorrhage**	**MACE**	**Major hemorrhage**
Sex	Male	0.14% (-0.80, 1.07)	1.22% (0.43, 1.99)	-0.75% (-2.21, 0.75)	1.32% (0.27, 2.37)
	Female	-0.78% (-1.29, -0.25)	0.01% (-0.39, 0.40)	-1.26% (-2.05, -0.46)	-0.49% (-1.04, 0.06)
Age	< 75	-0.24% (-0.69, 0.25)	0.25% (-0.19, 0.69)	-0.53% (-1.28, 0.28)	-0.16% (-0.73, 0.42)
	≥ 75	-0.59% (-1.43, 0.27)	0.44% (-0.13, 1.04)	-1.06% (-2.27, 0.18)	0.04% (-0.86, 0.91)
Diabetes	No	-0.25% (-0.74, 0.20)	0.28% (-0.11, 0.67)	-0.78% (-1.54, -0.03)	-0.20% (-0.74, 0.33)
	Yes	-2.23% (-3.48, -1.02)	0.46% (-0.58, 1.45)	-2.90% (-4.87, -0.95)	0.37% (-1.00, 1.71)
CVRF	0	0.17% (-0.54, 0.90)	0.82% (0.22, 1.43)	-0.13% (-1.20, 0.83)	0.62% (-0.17, 1.45)
	1	-1.13% (-1.84, -0.41)	-0.17% (-0.76, 0.40)	-2.03% (-3.13, -0.91)	-0.48% (-1.36, 0.37)
	2	-0.48% (-1.36, 0.43)	0.96% (0.23, 1.71)	-0.93% (-2.30, 0.44)	0.48% (-0.51, 1.48)

CVRF: cardiovascular risk factors

© 2026 Beydon M et al. *JAMA Network Open*.

eTable 11: Risk difference and relative risk of primary and secondary outcomes at 1-year according to sex

	1-year outcomes in women		1-year outcomes in men	
**Events**	**Absolute Risk Difference (%) (CI)**	**Relative Risk (CI)**	**Absolute Risk Difference (%) (CI)**	**Relative Risk (CI)**
**Primary outcome**	-0.78 (-1.29, -0.25)	0.79 (0.65, 0.92)	0.14 (-0.80, 1.07)	1.03 (0.80, 1.25)
All-cause mortality	-0.44 (-0.84, -0.03)	0.81 (0.64, 0.97)	-0.34 (-1.03, 0.31)	0.86 (0.58, 1.11)
Myocardial infarction	-0.19 (-0.41, 0.02)	0.67 (0.31, 0.99)	0.17 (-0.29, 0.62)	1.20 (0.54, 1.77)
Stroke	-0.25 (-0.51, -0.00)	0.75 (0.51, 0.97)	0.20 (-0.28, 0.69)	1.19 (0.66, 1.66)
**Major hemorrhage**	0.01 (-0.39, 0.40)	1.00 (0.77, 1.22)	1.22 (0.43, 1.99)	1.75 (1.12, 2.32)
Digestive bleeding	0.28 (-0.09, 0.65)	1.27 (0.86, 1.66)	0.98 (0.28, 1.68)	2.06 (1.03, 3.02)
Intracranial hemorrhage	-0.04 (-0.23, 0.15)	0.89 (0.37, 1.39)	0.43 (0.06, 0.80)	2.10 (0.15, 3.75)
Other hemorrhage	0.22 (-0.05, 0.48)	1.41 (0.80, 1.95)	0.92 (0.38, 1.47)	2.65 (1.01, 4.11)
**Net clinical benefit**	-0.40 (-1.05, 0.26)	0.92 (0.79, 1.05)	1.54 (0.38, 2.75)	1.28 (1.05, 1.52)

ASA: low-dose aspirin. Primary outcome is a composite outcome of all-cause mortality, myocardial infarction and stroke. Major hemorrhage is a composite of intracranial, digestive and other hemorrhage. Other hemorrhage is defined by a hospitalization with main diagnostic code for epistaxis, hematuria, hemothorax or intraocular hemorrhage.

Risk difference is given by the difference in probability in the low-dose aspirin arm and the control arm.

© 2026 Beydon M et al. *JAMA Network Open*.

eTable 12: Patients' characteristics of patients according to sex

	All patients<br>N = 14,528	Women<br>N = 10,371	Men<br>N = 4,118
Age at diagnosis	73.2 (8.7)	73.6 (8.7)	72.1 (8.7)
Polymyalgia rheumatica ICD code	3,362 (23%)	2,355 (23%)	999 (24%)
Length of hospital stay at diagnosis (days)	7.2 (5.7)	7.2 (5.7)	7.2 (5.7)
Comorbidities			
Diabetes	2,233 (15%)	1,480 (14%)	785 (19%)
Hypertension	8,263 (57%)	6,130 (59%)	2,170 (53%)
Dyslipidemia	5,239 (36%)	3,903 (38%)	1,363 (33%)
Cancer	2,012 (14%)	1,350 (13%)	673 (16%)
Dementia	602 (4.1%)	464 (4.5%)	144 (3.5%)
Tobacco use	1,634 (11%)	1,062 (10%)	584 (14%)
Obesity	574 (4.0%)	426 (4.1%)	150 (3.6%)
Chronic kidney disease	355 (2.4%)	246 (2.4%)	113 (2.7%)
Alcohol abuse	287 (2.0%)	148 (1.4%)	142 (3.4%)
Cardiac insufficiency	185 (1.3%)	132 (1.3%)	54 (1.3%)
History of major hemorrhage	553 (3.8%)	372 (3.6%)	180 (4.4%)
Treatments			
Frequent corticosteroid use	2,774 (19%)	2,083 (20%)	684 (17%)
Proton pump inhibitor at baseline	7,422 (51%)	6,232 (60%)	2,426 (59%)
Actual or past iron deficiency	2,978 (20%)	2,430 (23%)	581 (14%)
Tocilizumab	121 (0.8%)	157 (1.5%)	53 (1.3%)
Methotrexate	419 (2.9%)	355 (3.4%)	125 (3.0%)
Polymedication	4,301 (30%)	3,231 (31%)	1,059 (26%)
History of aspirin use	1,213 (8.3%)	863 (8.3%)	342 (8.3%)

© 2026 Beydon M et al. *JAMA Network Open*.

eFigure 1: Flow chart

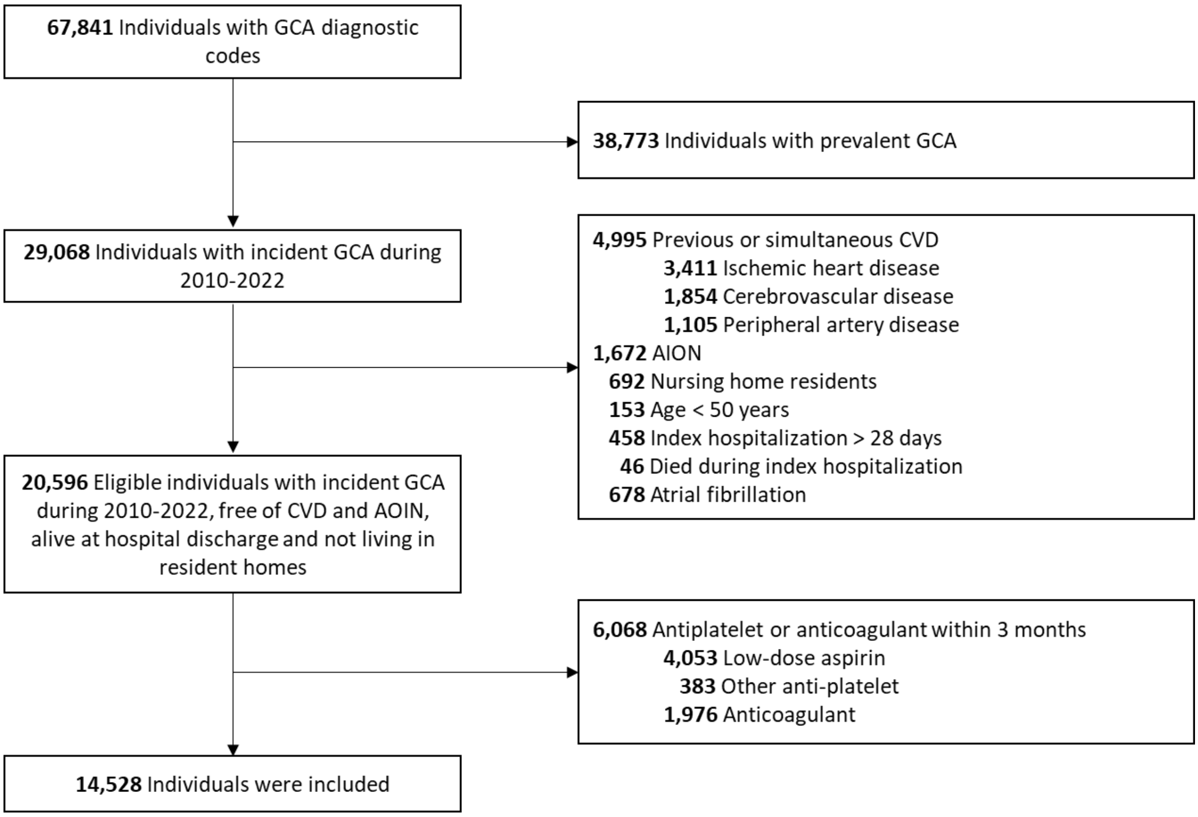

Legend: GCA: giant cell arteritis, CVD: cardiovascular disease, AOIN: anterior optic ischemic neuropathy

© 2026 Beydon M et al. *JAMA Network Open*.

eFigure 2: Standardized differences across treatment arms by the end of the grace period, before and after inverse probability of censoring weighting

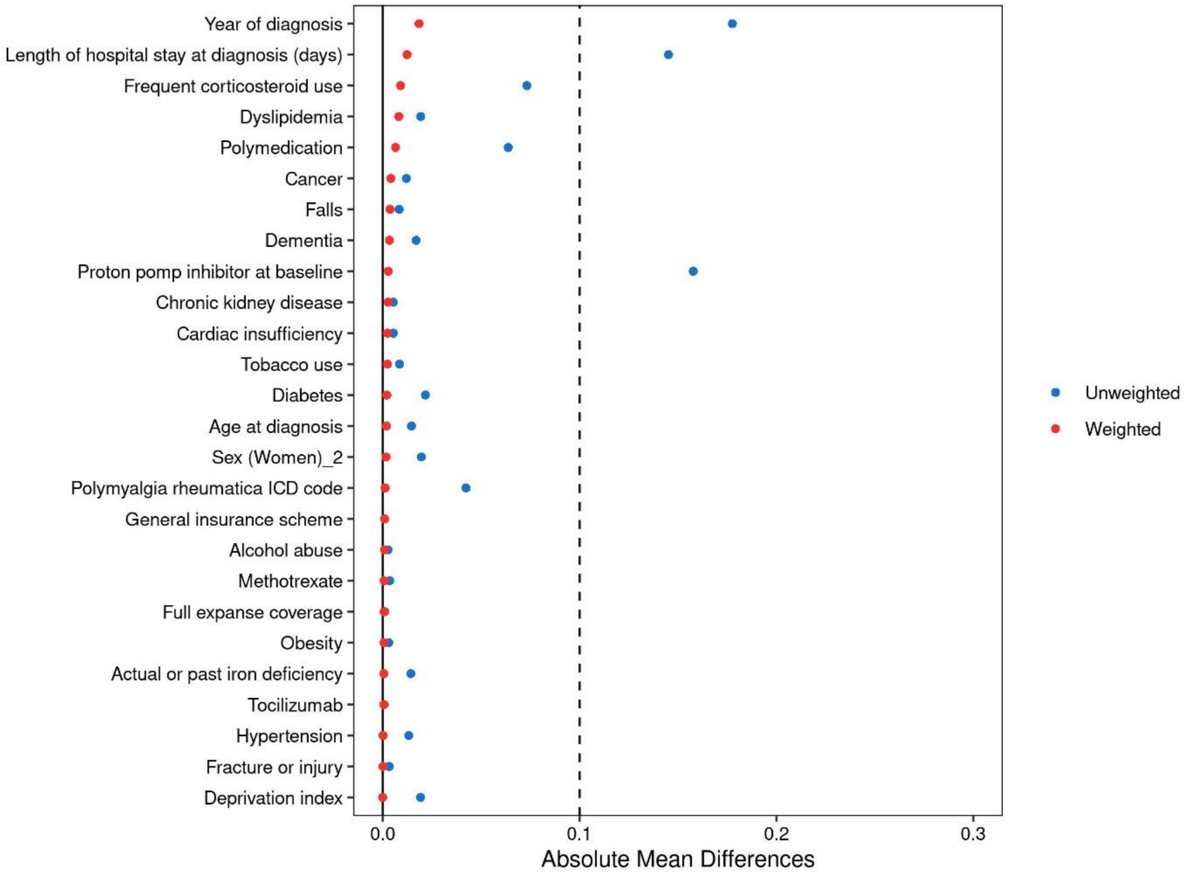

© 2026 Beydon M et al. *JAMA Network Open*.

eFigure 3: Proportion of patients initiating low-dose aspirin by year of GCA diagnosis

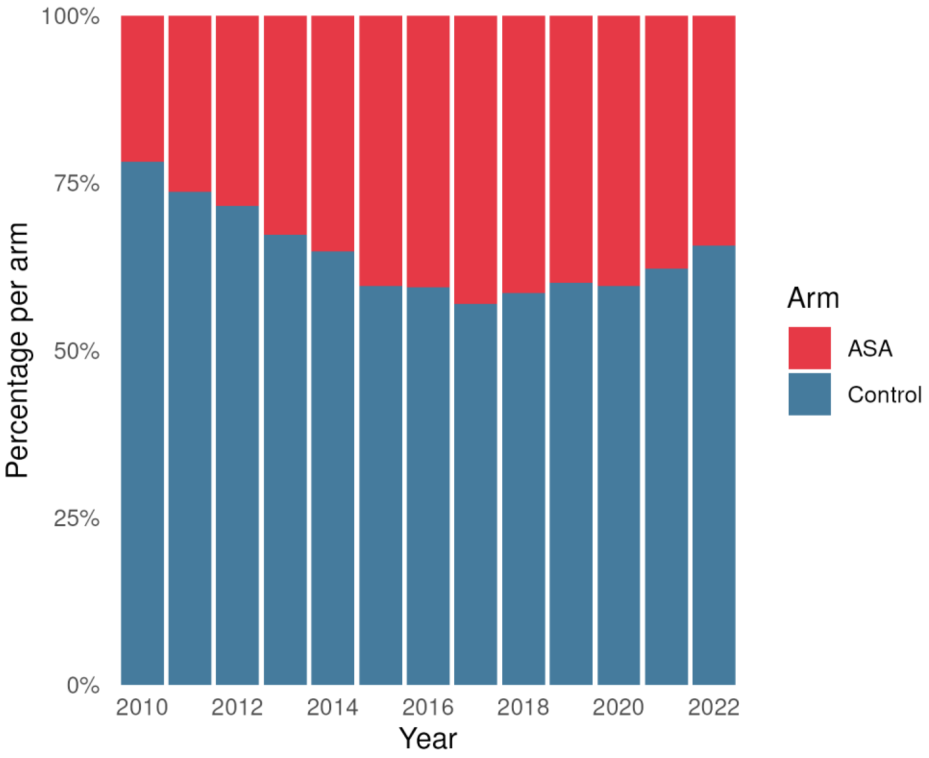

ASA: low-dose aspirin

© 2026 Beydon M et al. *JAMA Network Open*.

eFigure 4: Probability of deviating from assigned treatment arm after the grace period

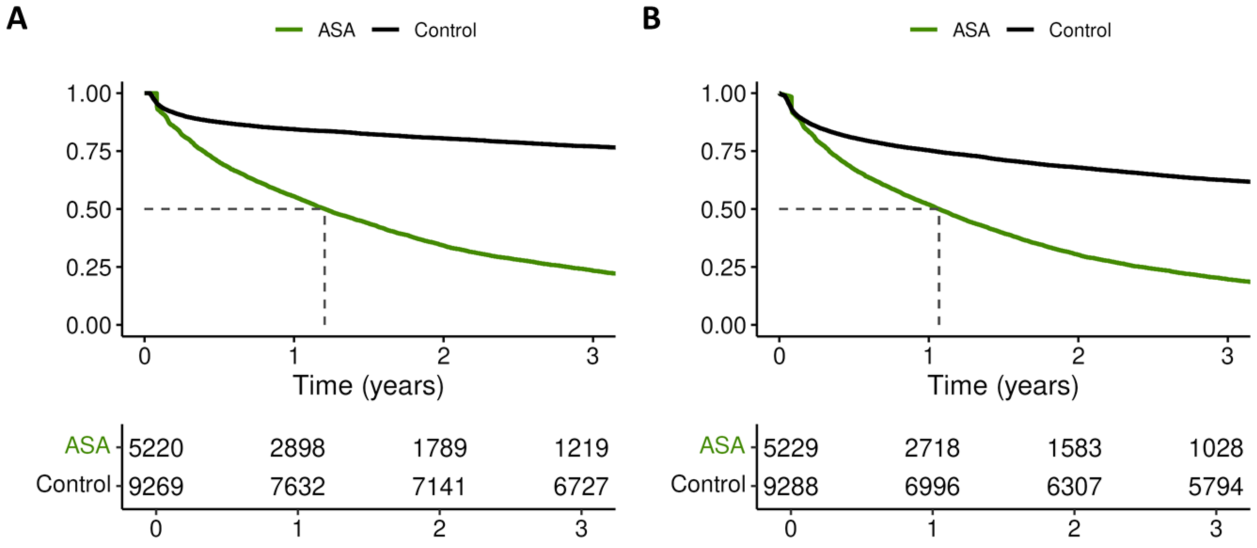

Censoring in case of introducing aspirin in the control arm or interrupting of aspirin in the control arm, unless otherwise indicated (Panel A). Further censoring in case of introducing of another antiplatelet or anticoagulant in any arm (Panel B). ASA: low-dose aspirin

eFigure 5.1: Cumulative incidence of secondary cardiovascular endpoints

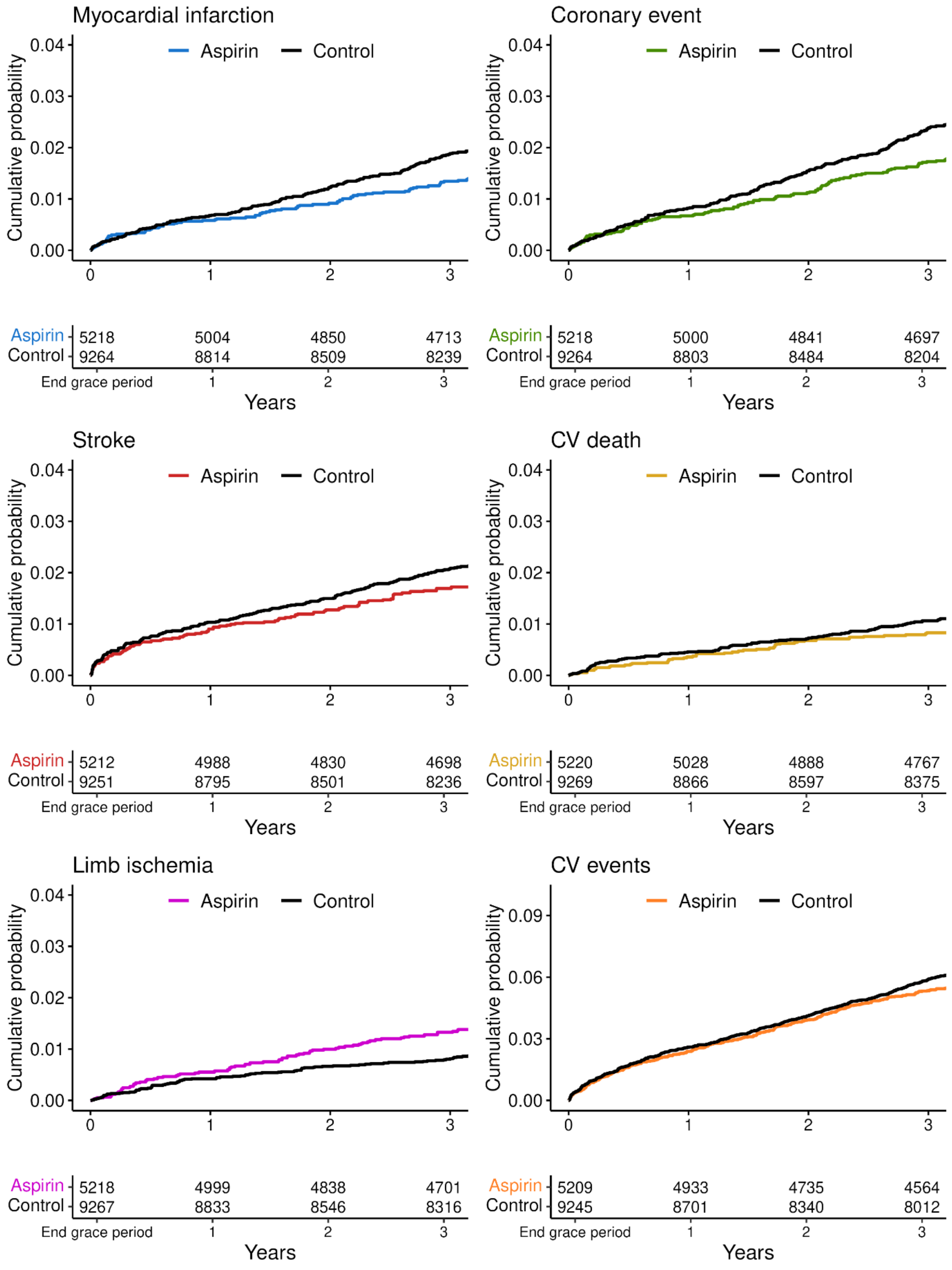

© 2026 Beydon M et al. *JAMA Network Open*.

eFigure 5.2: Cumulative incidence of hemorrhagic endpoints

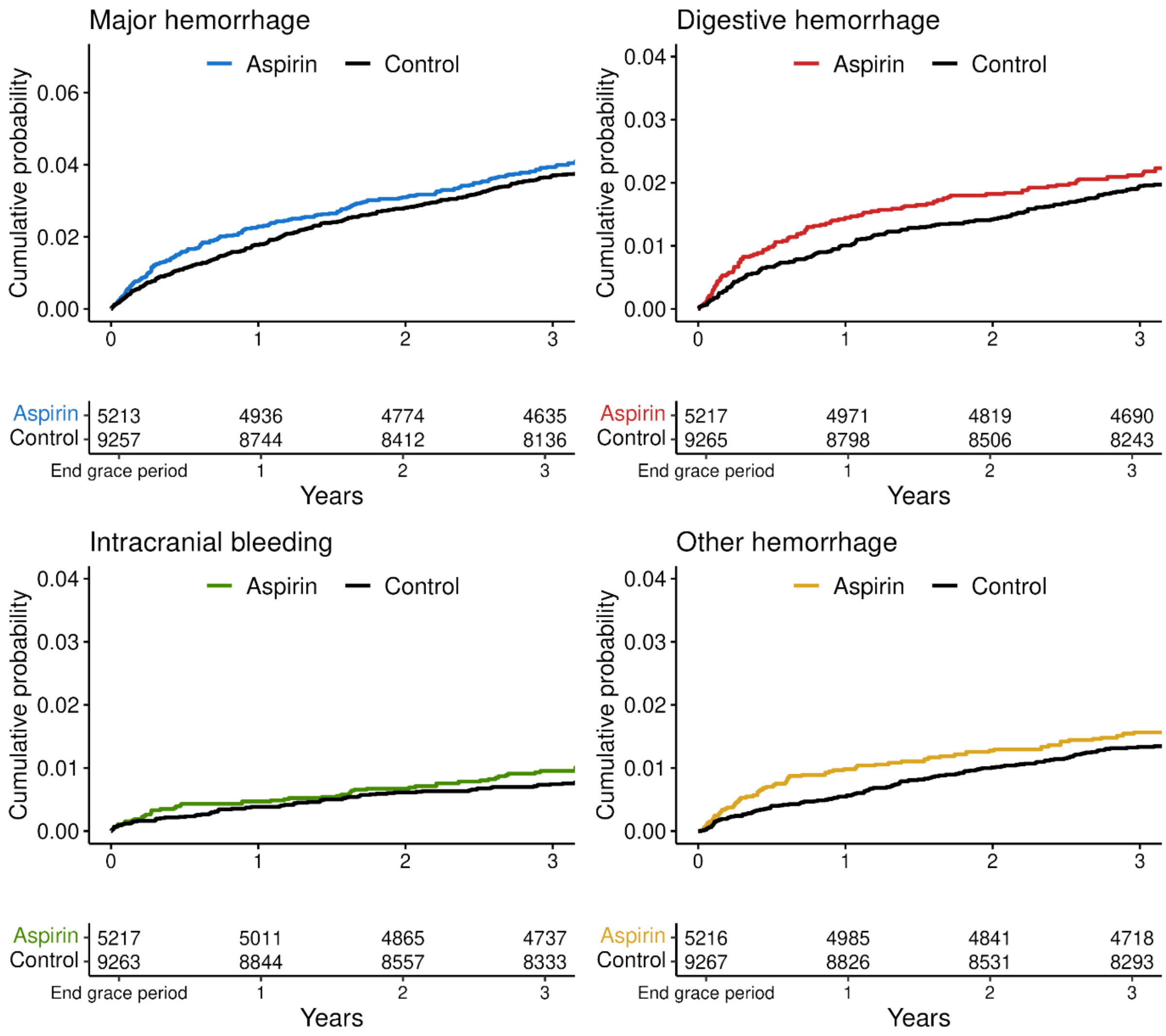

© 2026 Beydon M et al. *JAMA Network Open*.

eFigure 6: Cumulative incidence of main and secondary endpoints (A) and risk difference at pre-defined timepoints (B) in the per-protocol analysis

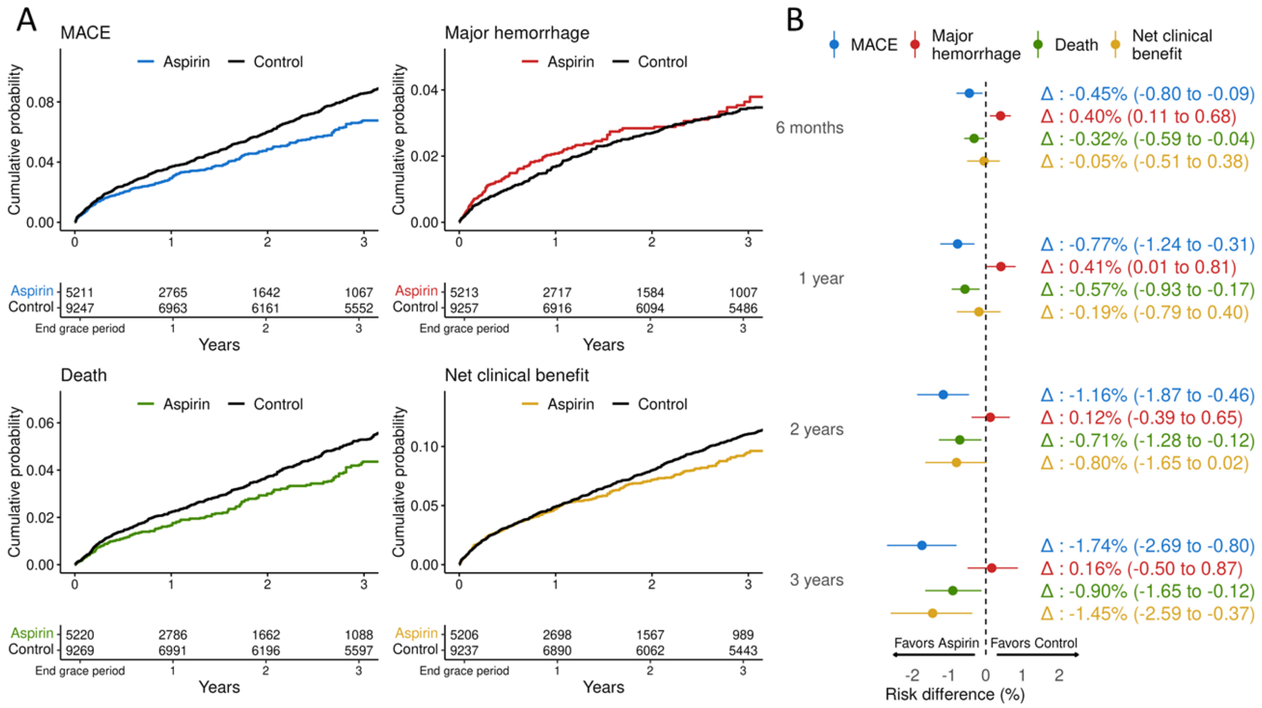

Legend: MACE : Major cardiovascular events (composite outcome of all-cause mortality, myocardial infarction and ischemic stroke). Major hemorrhage if a composite event of intracranial bleeding, digestive bleeding and hospitalization for other hemorrhage.
In this analysis, patients are further censored after grace period in case of aspirin or other antiplatelet/anticoagulant in the control arm, or aspirin interruption or introduction of other antiplatelet/anticoagulant in the aspirin arm.

© 2026 Beydon M et al. *JAMA Network Open*.

eFigure 7: Probability of MACE and major hemorrhage according to sex and age at Giant Cell Arteritis diagnosis

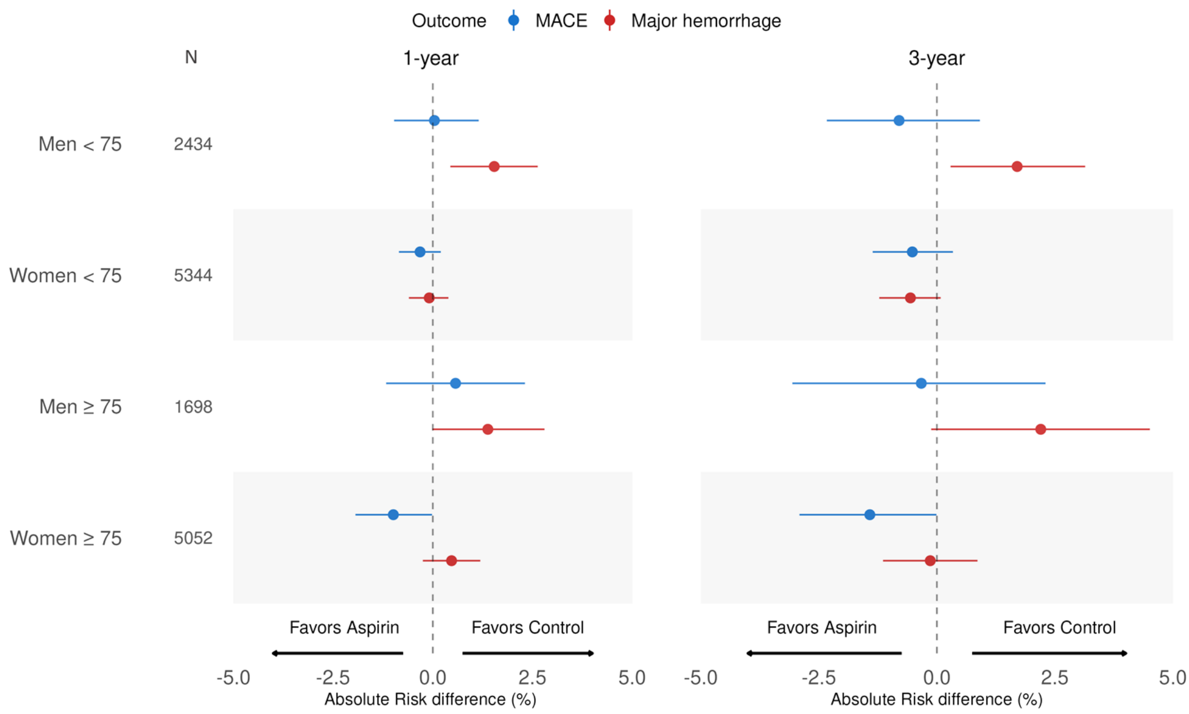

Legend: MACE : Major cardiovascular (composite outcome of all-cause mortality, myocardial infarction and ischemic stroke). Major hemorrhage if a composite event of intracranial bleeding, digestive bleeding and hospitalization for other hemorrhage. CVRF : cardiovascular risk factors. Risk difference is expressed as the difference in probability between the aspirin arm and the control arm.

© 2026 Beydon M et al. *JAMA Network Open*.

eFigure 8.1: Cumulative incidence of main and secondary endpoints according to sex

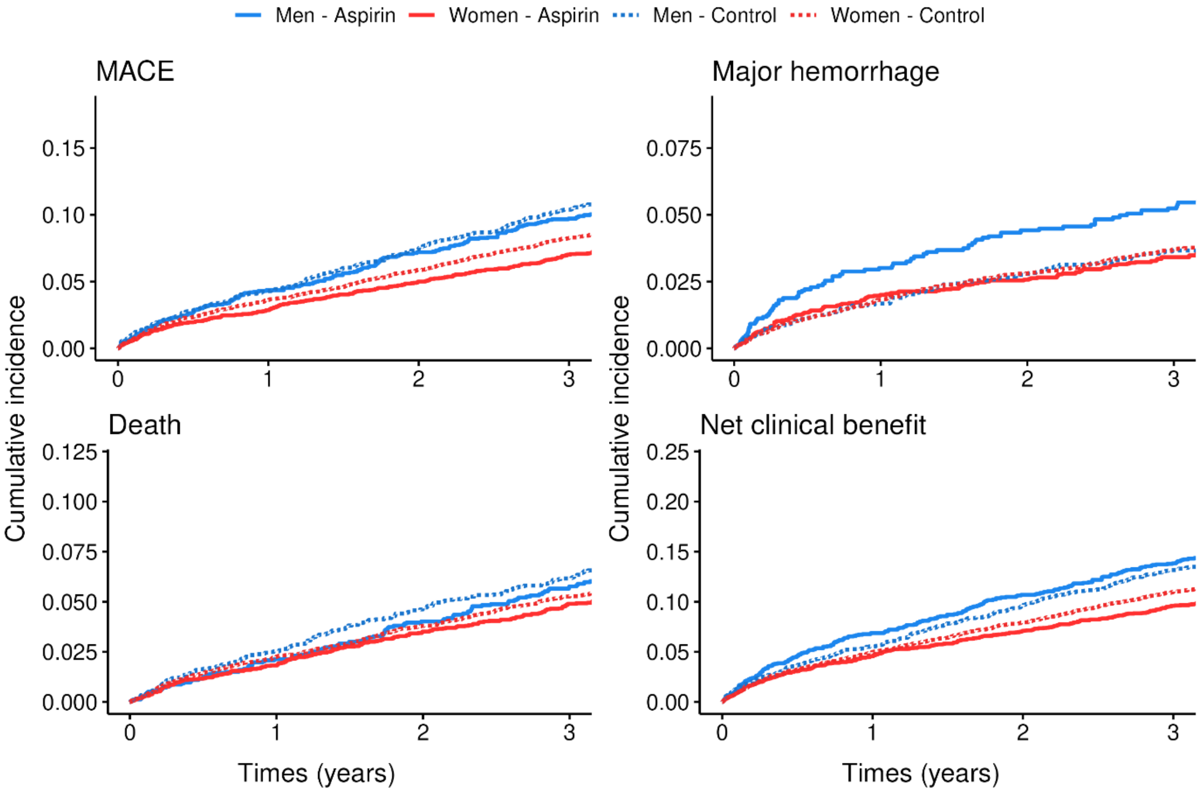

© 2026 Beydon M et al. *JAMA Network Open*.

eFigure 8.2: Cumulative incidence main and secondary endpoints according to sex

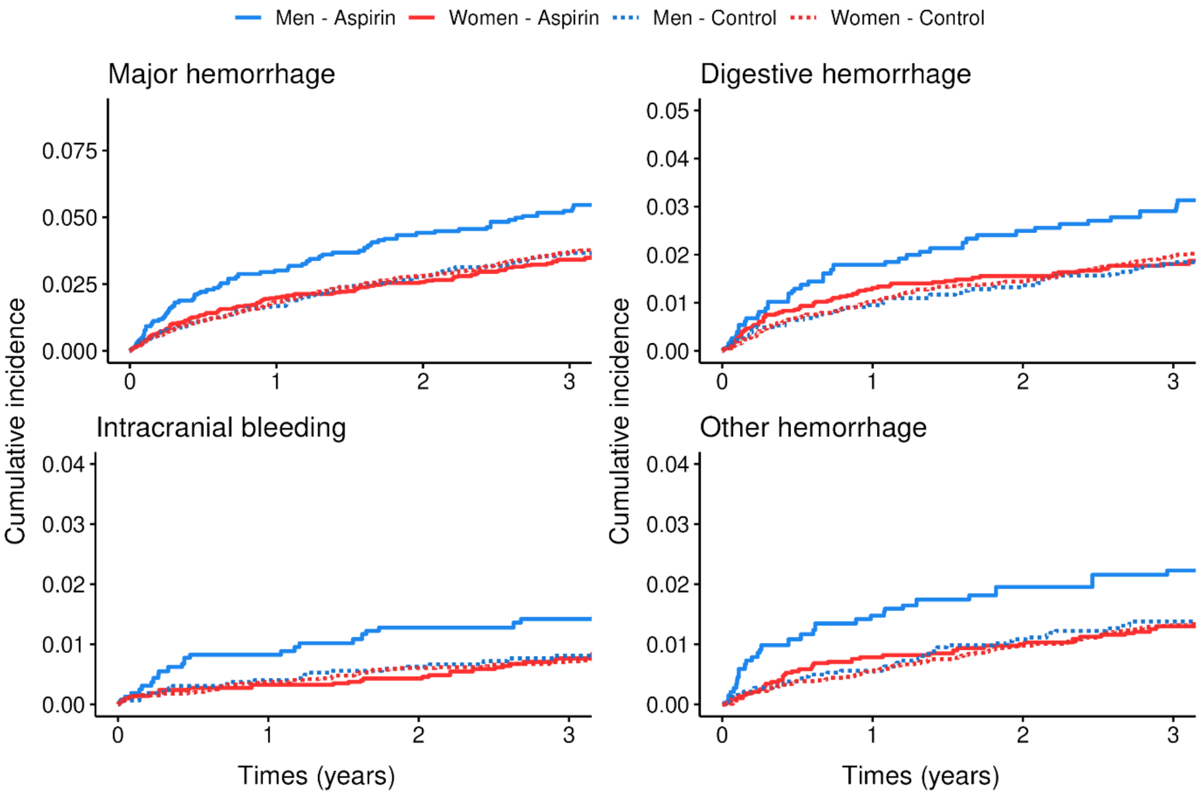

© 2026 Beydon M et al. *JAMA Network Open*.

eFigure 8.3: Cumulative incidence main and secondary endpoints according to sex

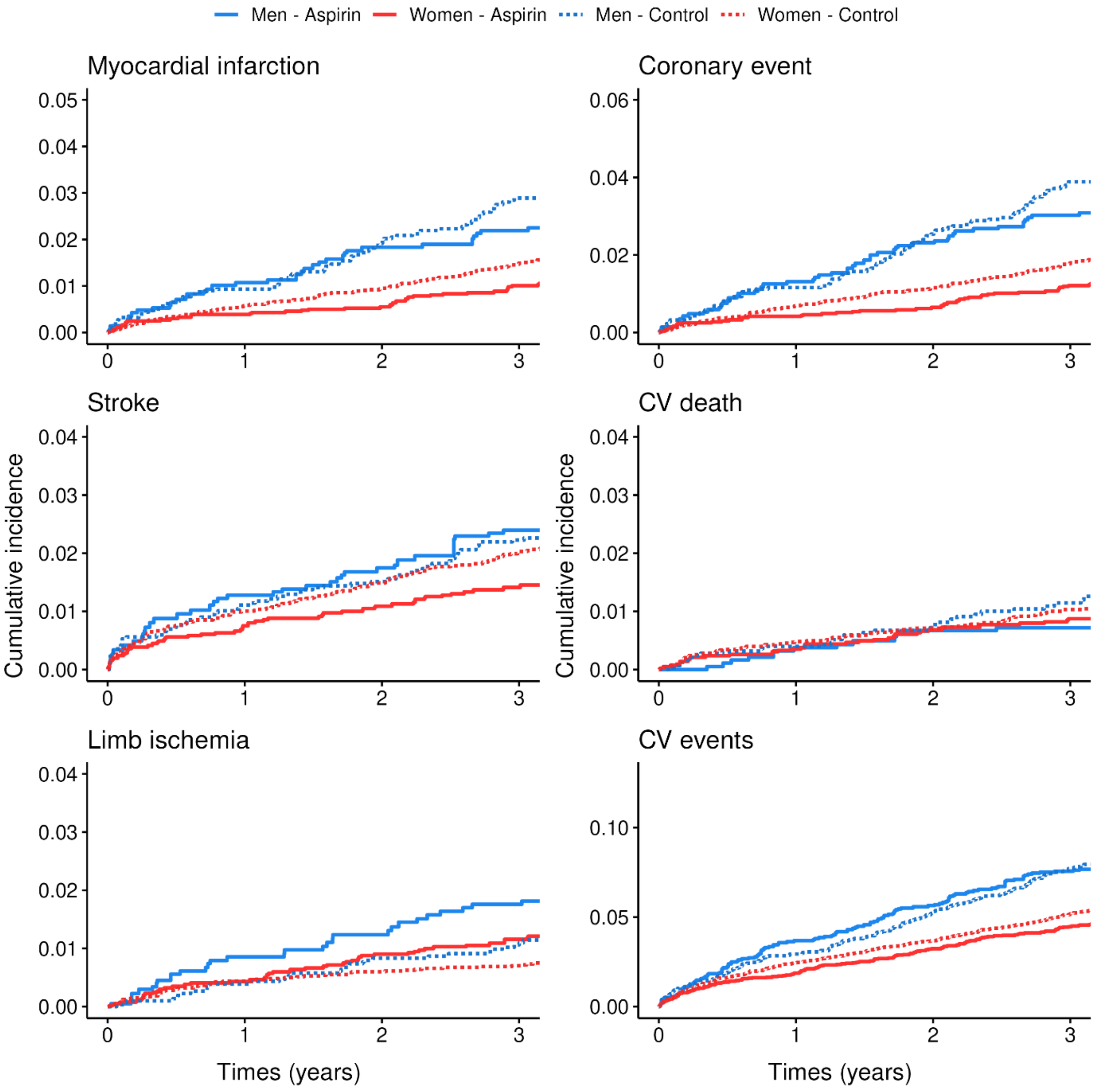

© 2026 Beydon M et al. *JAMA Network Open*.

eFigure 9: Cumulative incidence of main and secondary endpoints (A) and risk difference at pre-defined timepoints (B) after exclusion of patients with any history of low-dose aspirin deliverance

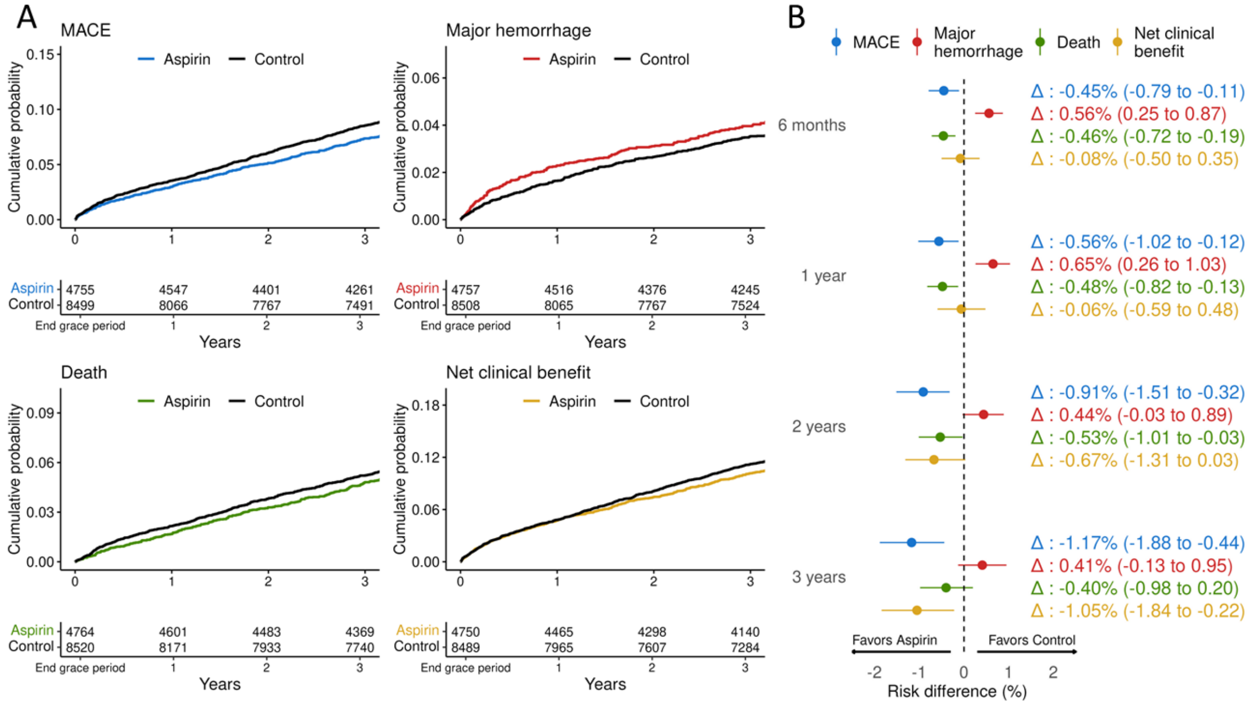

© 2026 Beydon M et al. *JAMA Network Open*.

eFigure 10: Cumulative incidence of main and secondary endpoints (A) and risk difference at pre-defined timepoint (B) after exclusion of patients with frequent steroid use or methotrexate or tocilizumab at baseline (sensitivity)

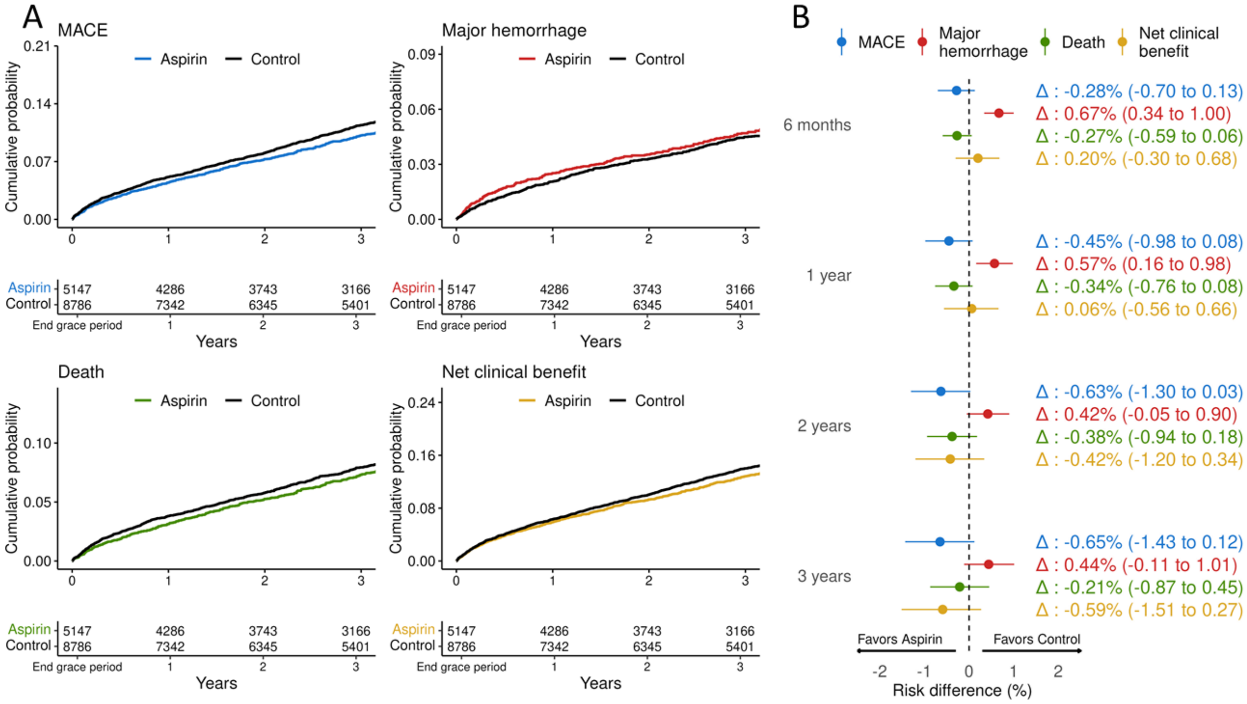

© 2026 Beydon M et al. *JAMA Network Open*.

eFigure 11: Cumulative incidence of main and secondary endpoints (A) and risk difference at pre-defined timepoint (B) after censoring patients without any steroid delivery at 30-day following index date

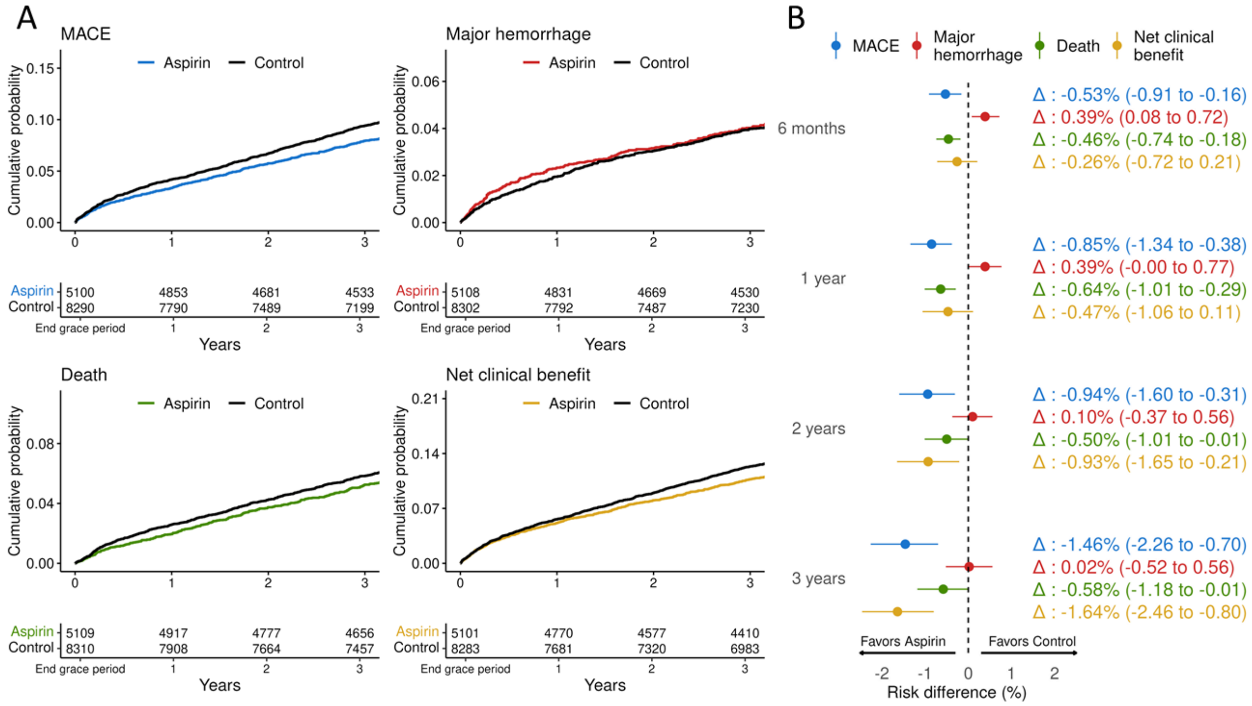

© 2026 Beydon M et al. *JAMA Network Open*.

eFigure 12: Cumulative incidence of main and secondary endpoints (A) and risk difference at pre-defined timepoint (B) with grace period of 7 days (sensitivity)

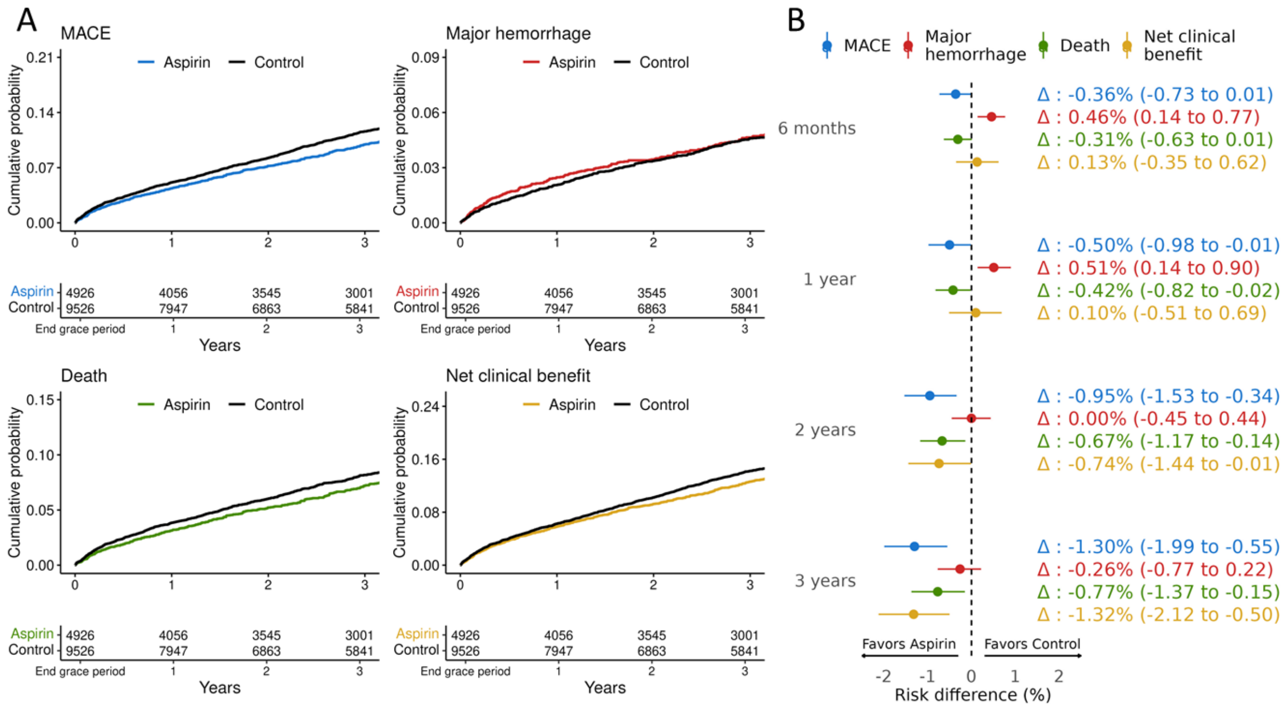

© 2026 Beydon M et al. *JAMA Network Open*.

eFigure 13: Cumulative incidence of main and secondary endpoints (A) and risk difference at pre-defined timepoint (B) within inclusions starting January 1st 2016

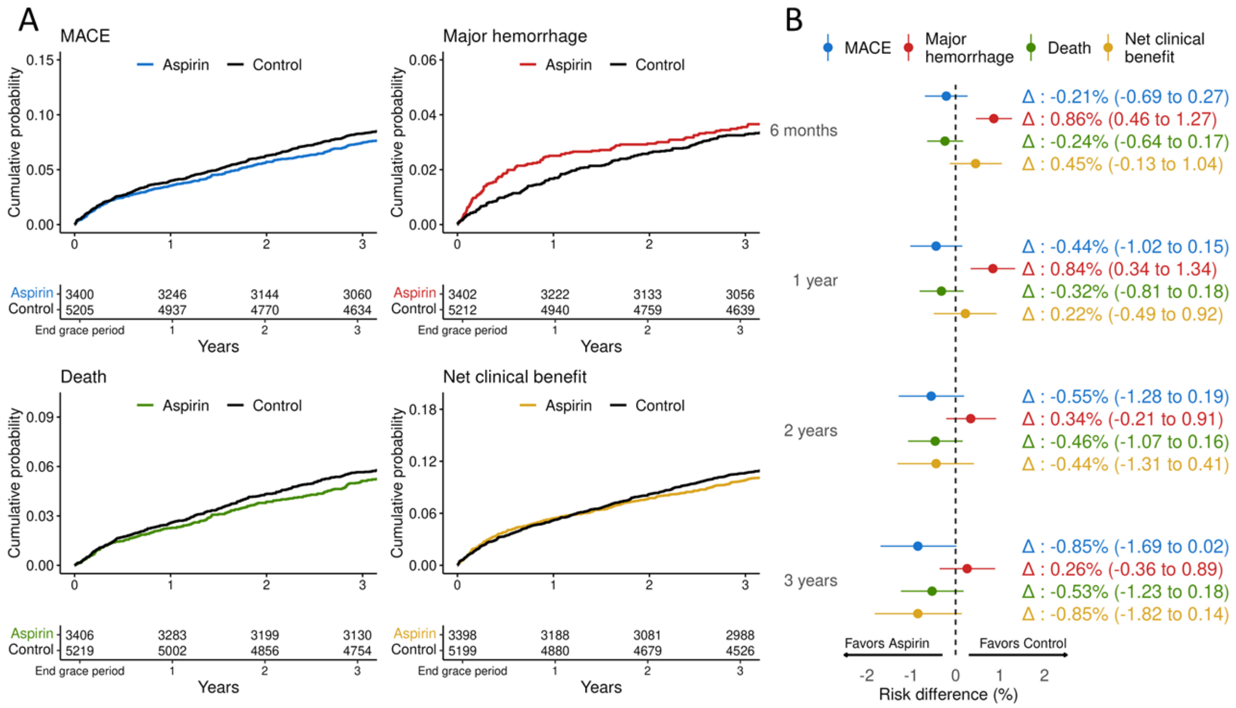

© 2026 Beydon M et al. *JAMA Network Open*.

eFigure 14: Cumulative incidence of infection and composite outcome of infection or death (A) and risk difference at pre-defined timepoints (B)

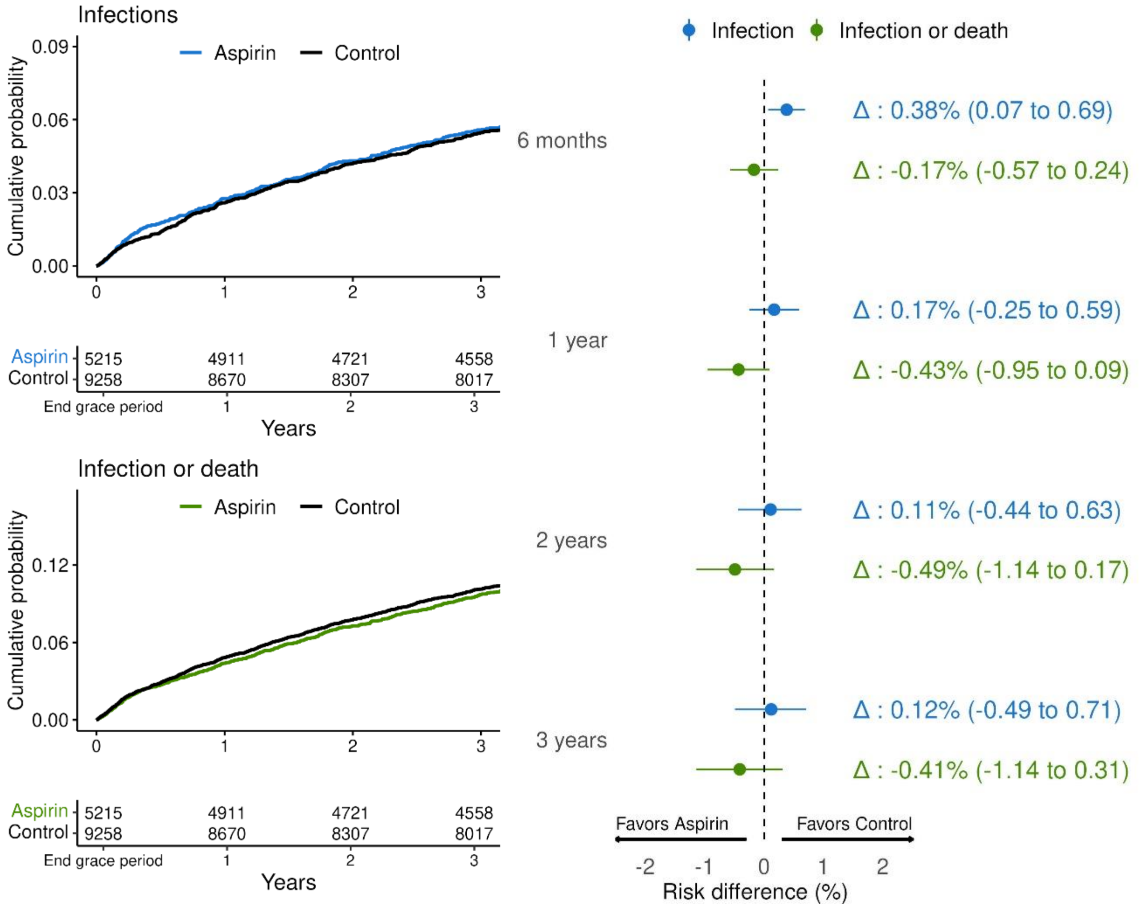

© 2026 Beydon M et al. *JAMA Network Open*.

eFigure 15: Cumulative incidence of main and secondary endpoints (A) and risk difference at pre-defined timepoint (B) according to laxative delivery during grace period

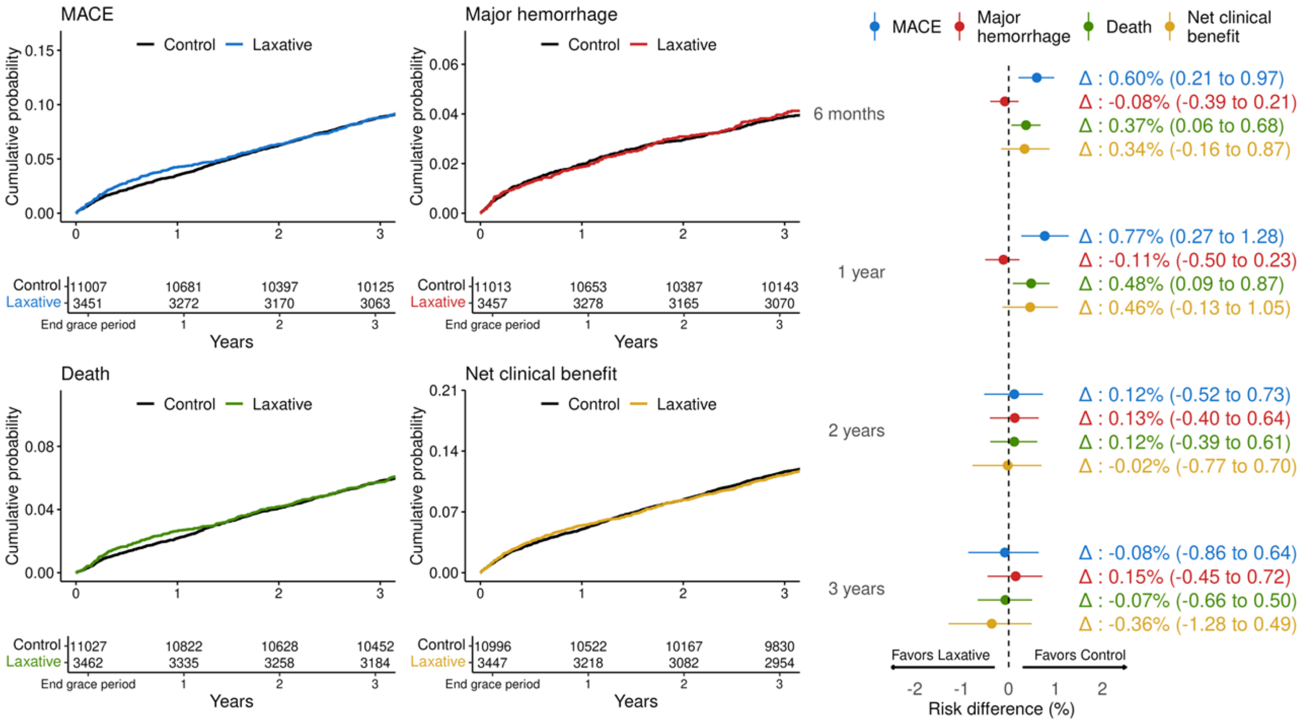

© 2026 Beydon M et al. *JAMA Network Open*.

eFigure 16: Difference in MACE and major hemorrhage in different subgroups according to laxative delivery during grace period

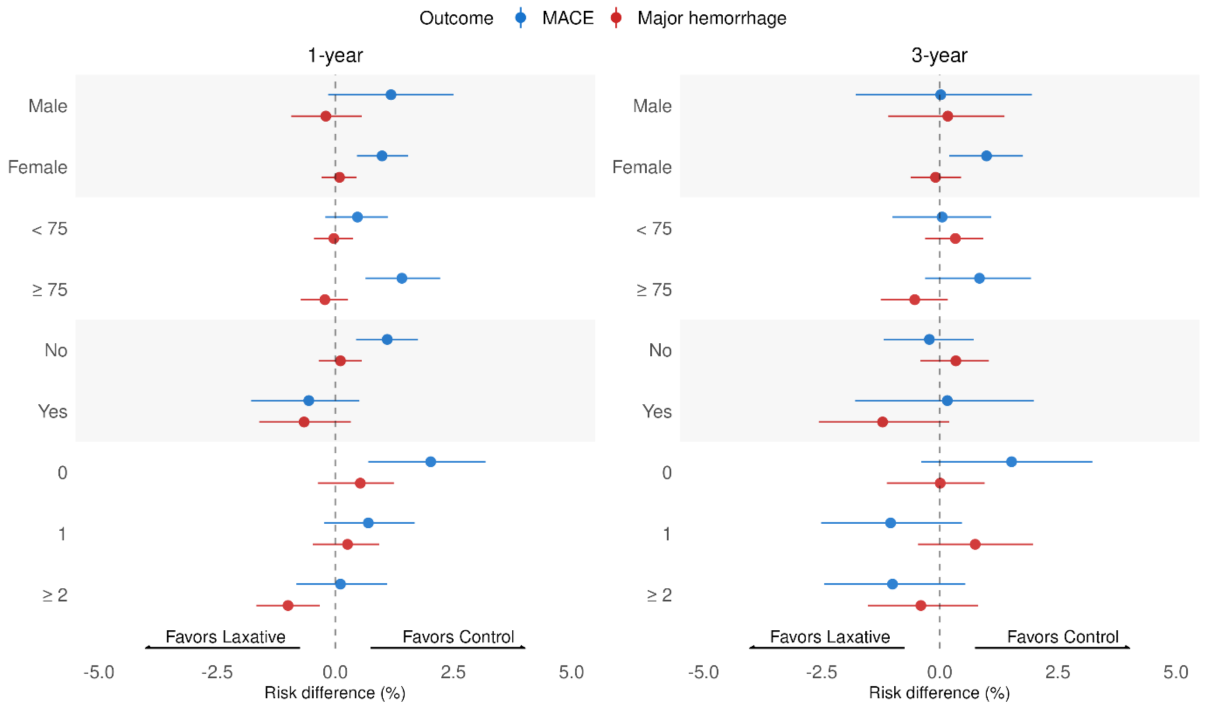

© 2026 Beydon M et al. *JAMA Network Open*.
