# Supplementary material for: Low-Dose Aspirin for Cardiovascular Disease Primary Prevention in Patients With Giant Cell Arteritis
Source: JAMA Netw Open. 2026 Apr 17;9(4):e266579. doi: 10.1001/jamanetworkopen.2026.6579 (PMC13090850; doi:10.1001/jamanetworkopen.2026.6579)
Supplement: Supplement 2. — Data Sharing Statement [file jamanetwopen-e266579-s002.pdf]

Data Sharing Statement

Beydon. Low-Dose Aspirin for Cardiovascular Disease Primary Prevention in Patients With Giant Cell Arteritis. *JAMA Netw Open*. Published April 17, 2026.
doi:10.1001/jamanetworkopen.2026.6579

Data
Data available: No

Additional Information
Explanation for why data not available: The data used in this study are not publicly available due to confidentiality and privacy restrictions. Researchers who meet the criteria for access to confidential data may contact the corresponding author to discuss potential access arrangements.
